# Developing a novel typology of unprofessional behaviours between healthcare staff: a best fit framework synthesis

**DOI:** 10.1186/s12913-025-13962-5

**Published:** 2026-01-24

**Authors:** Justin Aunger, Ruth Abrams, Russell Mannion, Aled Jones, Judy M. Wright, Johanna I. Westbrook, Mark Pearson, Jill Maben

**Affiliations:** 1 NIHR Midlands Patient Safety Research Collaboration, University of Birmingham, Birmingham, UK; 2 Department of Applied Health Research, College of Medicine and Health, University of Birmingham, Birmingham, UK; 3 School of Health Sciences, Faculty of Health and Medical Sciences, University of Surrey, Guildford, UK; 4 Health Services Management Centre, University of Birmingham, Birmingham, UK; 5 School of Nursing and Midwifery, Faculty of Health, University of Plymouth, Plymouth, UK; 6 School of Medicine, Faculty of Medicine and Health, University of Leeds, Leeds, UK; 7 Australian Institute of Health Innovation, Macquarie University, Sydney, New South Wales, Australia; 8 Wolfson Palliative Care Research Centre, Hull York Medical School, University of Hull, Hull, UK

**Keywords:** Unprofessional behaviour, Patient safety, Bullying, Interventions, Healthcare, Workforce

## Abstract

**Background:**

Unprofessional behaviours such as bullying, harassment, and microaggressions negatively affect patient safety and staff psychological wellbeing in healthcare systems globally. These behaviours do so by: (i) inhibiting health care professionals’ abilities to speak up to raise safety concerns; (ii) impairing team communication and individuals’ concentration; and (iii) promoting tolerance of bad practice. Unfortunately, there is little consensus in practice or academia about how these behaviours are defined. This can lead to an underestimation of the prevalence of these behaviours, inhibition of speaking up by victims and bystanders, and reduced accountability by those who enact these behaviours. We aimed to map definitions of unprofessional behaviours between staff to understand their similarities and differences and to develop a useful typology for theory-informed interventions.

**Methods:**

We used a six-step modified best-fit framework synthesis methodology to formulate our new typology, as a part of a wider realist review project. We employed a systematic approach to develop a framework for understanding UB. First, we identified relevant literature through a systematic search of Embase, CINAHL and MEDLINE databases (and more) (*n* = 146 sources). An initial framework outlining the dimensions of unprofessional behaviours was then constructed based on extracted definitions. Terms from included studies were then coded against this framework, with new dimensions introduced as needed to accommodate terms that did not align with existing categories. The resulting framework was refined iteratively and validated through stakeholder engagement, enhancing its relevance and validity.

**Results:**

We identified 37 behaviours drawing on 146 literature sources and found little consensus in how unprofessional behaviours between staff are defined in the academic literature. By collating definitions, we identified five dimensions inherent to unprofessional behaviours between staff namely: visibility; inherent frequency; whether they are highly targeted; if behaviours target protected characteristics (personal attributes that are legally safeguarded against discrimination in the UK and many other countries, such as race, sex or religion); if behaviours are physical; and if hierarchy is required. These dimensions enabled formulation of the typology with increased understanding of the differences between unprofessional behaviour types.

**Conclusions:**

We found that poor and inconsistent understanding of unprofessional behaviour could undermine interventions by inhibiting speaking up, enabling instigators to avoid accountability, and inhibiting ability to measure unprofessional behaviour and address it. Our typology provides a useful resource for academics, healthcare organisations, intervention architects, and individuals who are seeking to understand and clarify the range of unprofessional behaviours that may be encountered in healthcare settings.

**Supplementary Information:**

The online version contains supplementary material available at 10.1186/s12913-025-13962-5.

## Background

The way healthcare staff treat patients and the way staff treat each other are often at odds. Poor interactions with between staff can take the form of ‘unprofessional behaviours’ (UBs) such as bullying, harassment, microaggressions, rudeness, incivility, or micromanagement [1]. Real-life examples reported by staff include (but are not limited to) “*aggressive ‘over-ruling’ and ‘eye-rolling’ type behaviour when expressing clinical concerns about patient conditions*” or being “*yelled at by a doctor after being falsely accused of not doing my job*” [2]. Based on our work analysing and coding definitions of multiple types of UB, we have defined UBs as “*Any interpersonal behaviour by staff that causes distress or harm to other staff in the healthcare workplace*” [1]. Such behaviours can be due to many factors including: working in a high pressure environment, a lack of resources, and opaque organisational processes that drive conflict [3]. UBs are important to address because research has frequently highlighted their significant impact on staff wellbeing and patient safety [3, 4]. For example, studies suggest that 40% of staff report moderate or major impacts on wellbeing after experiencing UB [4], and have linked this to risk of burnout and desire to leave an organisation, with increasing effects according to the degree of exposure [5].

Given the range of behaviours, understanding what constitutes UB varies significantly between individuals and organisations, and this can affect how they are measured and addressed. Researchers investigating UBs between staff have previously highlighted the impact of these definitional and measurement issues, stating that “*the absence of a comprehensive descriptive framework capturing and cataloguing these behaviours make identification, seeking assistance and intervention difficult*” [6]. Similarly, guidance for the development of complex interventions from the United Kingdom’s (UK) Medical Research Council highlights the importance of properly understanding “*the range of behaviours targeted*” by interventions [7].

## Problems stemming from inconsistent understanding

Inconsistent understanding of what may constitute UB can affect the ability of health care organisations to address it in three main ways. Firstly, ambiguous or ill thought-through use of language when measuring UB through surveys or instruments can misrepresent and thereby understate the prevalence of UB [4]; secondly, lack of certainty about what is unprofessional can inhibit speaking up by staff [8]; and, thirdly, exploiting ambiguity can enable instigators to get away with behaving unprofessionally [1, 9]. All of these can undermine intervention development and effectiveness.

### Understating prevalence of unprofessional behaviour

When an organisation tries to measure the prevalence of UB and its potential impact, it is important to measure all behaviours that can have a negative impact. The most common type of UBs encountered in a healthcare setting are rudeness, humiliation, and ostracisation, rather than bullying, abuse, or harassment [4, 10]. However, surveys such as the National Health Service (NHS) Staff Survey most commonly measure the latter [4, 11]. Despite the focus on behaviours perceived as more ‘severe’ such as bullying, harassment and abuse, even general rudeness has been shown to have serious implications for patient safety through inhibiting sharing of clinically important information, and for staff wellbeing [4, 11]. For example, a study in Australia by Westbrook et al. investigated UB amongst hospital staff using the Longitudinal Investigation of Negative Behaviour (LION) survey [4]. This survey lists 26 questions relating to different types of UB, ranging from “Being ignored or excluded” and “Excessive monitoring of work”, to “Demands for sexual favours”. They found that as many as 94% of staff at hospitals reported experiencing at least one ‘unprofessional behaviour’ from a staff member in the past year [4]. Indeed, the most common behaviours identified were having opinions ignored, and being spoken to rudely – behaviours not adequately captured by other surveys such as the NHS Staff Survey [4]. This demonstrates that only asking about “bullying, harassment, and abuse” can underestimate the true prevalence of wider UB. Misrepresentation of the extent and prevalence of these behaviours could result in interventions which fail to target widespread problematic behaviours such as general rudeness in communication, focus instead on rarer behaviours such as bullying, and undersell the size and urgency of the problem to be addressed [3].

### Inhibition of speaking up

The lack of definition can also affect employee voice. Staff might not speak up if it is unclear to them whether they experienced something unprofessional or inappropriate. For example, one study has stated that: “*those who are exposed to [negative workplace behaviour], including new graduate nurses, senior nurses, and nurse unit managers, would have reported [the instigator] based upon their own understanding of these behaviours*” [8]. Studies that present example vignettes of UB to both junior and senior staff have also shown that there are significant differences in perceptions between junior and senior staff (e.g. between students and clinicians) in perception of whether behaviours are UBs or not [12, 13].

Certain behaviours, such as microaggressions, are also more ambiguous in their intent but nonetheless can still cause harm. One definition of microaggressions from our literature is “*stunning and automatic acts of disrespect arising from unconscious attitudes inflicted by the culturally dominant groups*” [14]. An example may include sexist and/or racist behaviours such as “*when a junior male doctor is addressed by staff, overlooking the senior female doctor standing next to him*” [15]. This may allow instigators to go unnoticed, and their behaviours to go unaddressed, thus allowing such behaviours to become culturally dominant and normalised. There is therefore a need for organisations to clarify which behaviours are and are not acceptable in an organisation’s definitions of UBs and code of conduct and thus help to improve staff speaking up [16].

### Instigators can ‘muddy the waters’ of accountability

Ambiguity can also result in instigators avoiding sanctions for acting poorly, enabling such behaviours to become increasingly normalised. For example, after engaging in a microaggression, an instigator can plausibly claim ignorance about whether their behaviour was unprofessional. This issue may stem from more systemic origins, such as who writes organisational policies and which people are in positions of power (for example, in globalised minority countries most of whom are white, non-disabled, heterosexual etc.) [9]. This can impair organisations’ abilities to hold staff to account for such behaviours, can create ambiguity in relation to an organisation’s code of conduct, and can further damage culture [16]. Instigators are less likely to avoid accountability for poor behaviour if there is a more consistent understanding about what is acceptable.

### Ambiguity in the literature

The ambiguity around terms and what constitutes UB is problematic because it also extends to the academic literature. There is often little agreement regarding how terms such as bullying, incivility, harassment, or any other UB are defined. [1]. Definitional issues around UB inhibits academic consensus and thus research to design interventions to address these behaviours [1].

To work towards addressing these issues, we aimed to clarify the range of UB types, their dimensions, and produce a framework in the form of a typology for practitioners and researchers seeking to address UB.

## Methods

### Framework synthesis

We developed a typology to understand the dimensions of UBs and situate behaviours within it based on behavioural commonalities and differences [1] during a sub-study within a wider realist review [1, 3, 16, 17]. To do this, we used a modified best fit framework synthesis methodology to identify literature and form the typology [18, 19]. Framework synthesis is a wider group of methods that are suitable for use to “*develop theory, explore mid-range theory and develop logic models*” – and this extends to “*simple taxonomy development*” [20]. Framework synthesis involves development of a novel theory, typology, or framework, and enables the use of pre-identified themes or concepts as coding categories from the outset, while also allowing new themes or concepts to emerge through inductive analysis of data [21]. Its epistemological grounding as a method varies from realist-positivist to constructivist-interpretive; however, we approached this from a critical realist perspective, whereby we assumed an underlying reality that can be understood through structured frameworks [20].

**Fig. 1:**
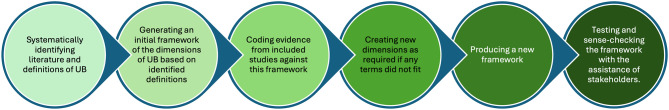
Six-step modified best fit framework synthesis method for generating the typology

Our method involved a six-step process (Fig. 1) of (1) systematically identifying literature and definitions of UB, (2) generating an initial framework of the dimensions of UB based on identified definitions, (3) coding evidence from included studies against this framework, (4) creating new dimensions as required if any terms did not fit, (5) producing a new framework and (6) testing and sense-checking the framework with the assistance of stakeholders. The only modification to the traditional series of steps in best fit framework synthesis is that we used ‘dimensions’ emerging from identified definitions themselves to form our initial a priori framework, rather than drawing on an established existing framework. This was because there were no identified appropriate a priori established typologies of UB in healthcare.

### Systematic search

We systematically searched Embase, CINAHL and MEDLINE databases for published literature, and HMIC, NICE Evidence Search, Patient Safety Network, Google and Google Scholar databases and NHS Employers and NHS Health Education England websites for grey literature. Supplementary File 1 outlines the full search syntax and details. As part of the wider realist study, searches were conducted in November 2021 and updated in December 2022.

We included studies which were: (1) of any design, (2) taking place in acute, critical and emergency healthcare settings, (3) talk about UB between healthcare staff rather than patient to staff or staff to patient, (4) and in English only.

### Data analysis

We started by collating and coding all the different terms identified in the included literature sources and all the ways in which these were defined by authors. First, we extracted definitions as we encountered them across the literature sources according to the term to which they referred. These definitions were then read and re-read to extract dimensions that were common across them. For example, we found that bullying was frequently defined as requiring a power hierarchy between the instigator and the victim. Similarly, we found that some behaviours were overt, and others were covert – which led to ‘visibility’ as a dimension. We therefore extracted these as common dimensions of definitions of UB. We then revisited the definitions of UB identified earlier and re-coded the terms identified against the dimensional framework according to how the behaviours were most commonly defined in the literature. Coding was performed in NVivo 12 software. For example, bullying was most frequently defined as being visible, being frequent rather than one-off, requiring a power hierarchy, and being highly targeted at one person. Once all behaviours were situated within one or more dimensions common across UBs, we were able to present these in a Venn-style diagram.

### Stakeholder involvement

To ‘sense-check’ the typology, we presented this typology to our stakeholder group on five occasions for comment, discussion and refinement. These groups consisted of expert academics, healthcare staff with experience of UBs, and patient and public members. Stakeholder feedback was integrated through a structured process: (1) presenting the draft framework to stakeholders for refinement; (2) documenting proposed modifications; (3) conducting purposive analyses to assess the validity of suggestions that deviated from the initial framework; (4) discussing discrepancies within the research team to reach consensus and determine appropriate actions; and (5) presenting the revised framework to stakeholders for further validation, for example, through “you said, we did” summaries at the beginning of each of five stakeholder meetings.

These five two-hour meetings took place from Jan 2022 to Mar 2023. Refinements made to the typology by stakeholder involvement included the initial suggestion of the dimension of ‘visibility’ – which we later added to the typology after analysing included definitions. After initial coding, they also helped query and test the position of specific behaviours in the final figure, and steered us away from including ‘severity’ as a dimension due to its experiential nature.

## Results

### Study selection

**Fig. 2:**
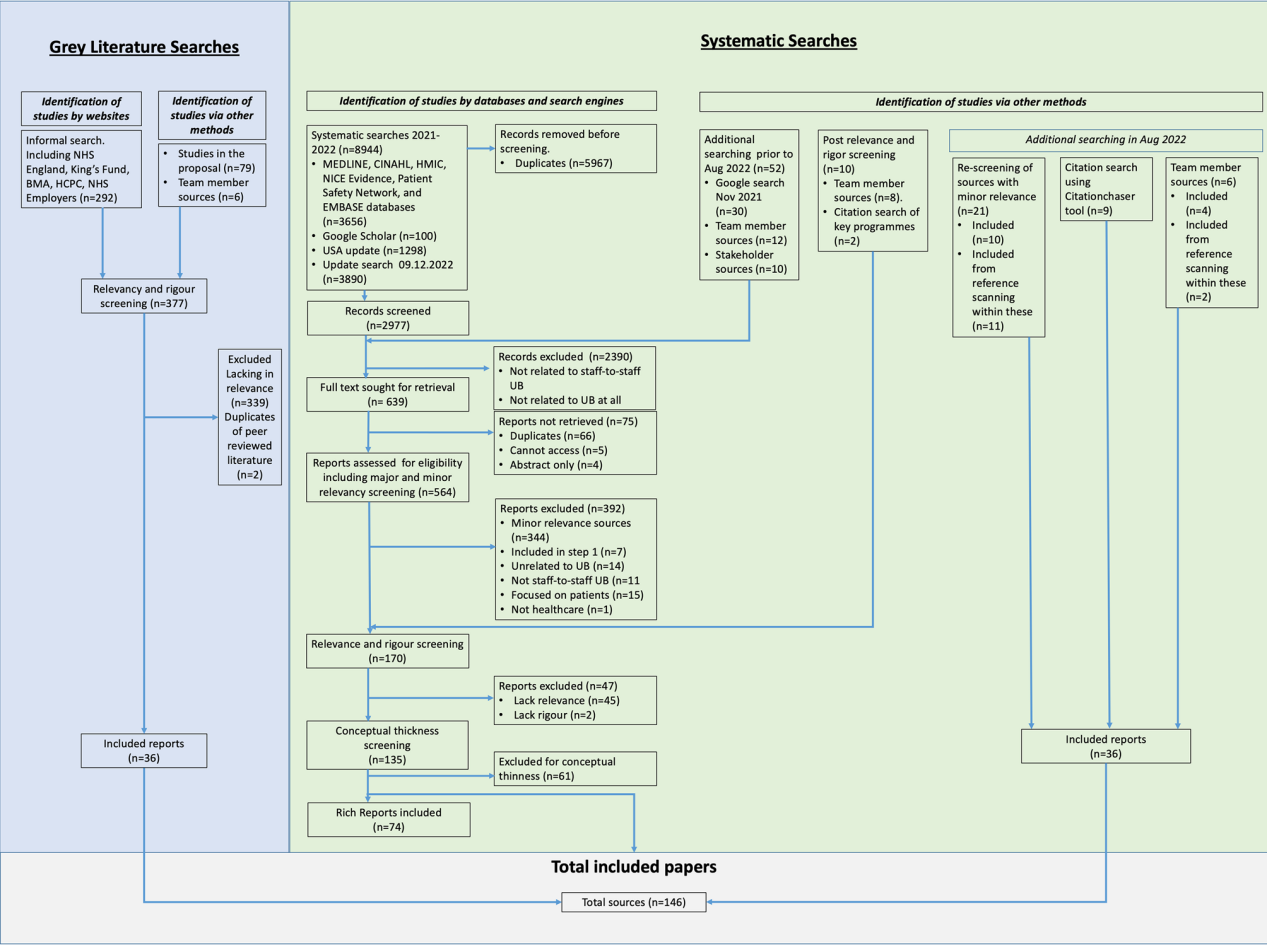
PRISMA diagram

Our systematic searches identified 8,944 records, reducing to 2,977 after de-duplication (*n* = 5,967). We also added reports identified via Google, team members, and stakeholders (*n* = 62). After application of inclusion and exclusion criteria, full text and conceptual richness screening, and relevancy and rigour screening, 146 reports were included. Supplementary File 2 outlines the characteristics of all 146 included sources. Full screening and searching results are reported elsewhere [16]. Figure 2 depicts the results of the grey and systematic literature searches.

### Identified behaviours and definitions

Our coding process identified 37 different terms defined in the literature including terms such as incivility, horizontal violence, lateral violence, disruptive behaviour, microaggressions, mobbing, negative workplace behaviour, and many more. Full extraction of these definitions and all 146 included references are presented in Supplementary File 3.

We also identified that there was often little agreement regarding how terms such as bullying, harassment, or any other UB are defined in the literature. This often manifested in disagreements regarding the dimensions inherent to particular behaviours/terms (e.g. whether incivility is inherently frequent or can be a one-off occurrence). To illustrate this, we collated examples of contrasting definitions and key discrepancies in Table 1. This highlights and confirms the issues we identified earlier, with the potential for these discrepancies to inhibit speaking up, enable instigators to avoid accountability, and reduce ability to measure and understand UB.

**Table 1:** Examples of varying definitions for the same behaviour identified in the literature. Reproduced with permission under CC-BY 4.0 license from Aunger et al. [1]

Term	Definition 1	Definition 2	Key discrepancy
Bullying	“*a form of harassment which involves persistent, intimidating behaviour, usually by a supervisor toward an employee*.” [22]	“*repeated exposure to person-, work-, and intimidation-related negative acts such as abuse, teasing, ridicule, and social exclusion over a period of time in the workplace.*” [23]	Definition 1 suggests a hierarchy must be present but definition 2 does not.
Lateral violence	“*Lateral violence (LV) is described as behaviour demonstrated by nurses who overtly or covertly direct dissatisfaction toward those less powerful than themselves and each other.*” [24]	“*Lateral violence is any repetitive behaviour among peers that is considered offensive, abusive, or intimidating by the target*.” [25]	Definition 1 suggests lateral violence can be towards those lower on the hierarchy, whereas hierarchy is not mentioned in definition 2 and suggests incivility must be frequent/repeated.
Incivility	“*subtle behaviours not intended to harm anyone but contrary to workplace standards.*” [26]	“*… repeated offensive, abusive, intimidating, or insulting behaviour, abuse of power, or unfair sanctions that make recipients upset and feel humiliated, vulnerable, or threatened, creating stress and undermining their self-confidence.*” [27]	Definition 1 suggests that incivility encompasses more subtle behaviours but definition 2 suggests they are not at all subtle.
Disruptive behaviour	“*The American Medical Association’s Council on Ethical and Judicial Affairs defines disruptive behaviour as behaviour that “tends to cause distress among other staff and affect overall morale within the work environment, undermining productivity and possibly leading to high staff turnover or even resulting in ineffective or substandard care*.” [28]	“*… we define disruptive behaviour as constituting the following three criteria: a) interpersonal (i.e., directed toward others or occurs in the presence of others); b) results in a perceived threat to victims and/or witnesses; c) violates a reasonable person’s standard of respectful behaviour*”.[29]	Definition 1 suggests disruptive behaviour includes passive-type behaviours that undermine productivity, whereas definition 2 suggests it must be interpersonal.

### Dimensions of behaviours and discrepancies in the literature

Step 2 of our process involved identifying key dimensions, or commonalities, found across definitions of UBs. The dimensions we identified were: inherent frequency of the behaviour, intentional targeting (or not) of the behaviour, visibility of the behaviour to others, requirement of power hierarchy, whether the behaviour extends to physical contact, and whether the behaviour exclusively targets protected characteristics (e.g. gender, age, disability, race).

### Presenting the typology

**Fig. 3:**
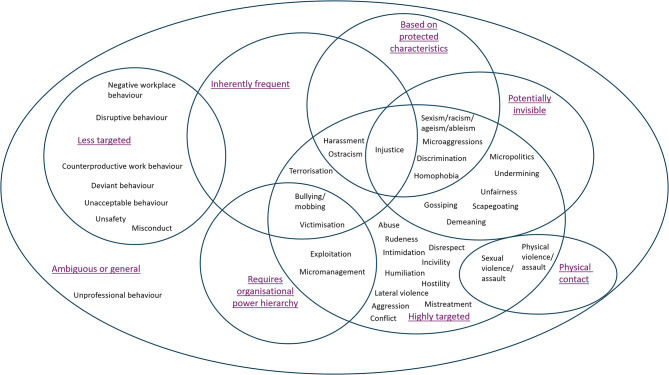
Typology of unprofessional behaviours. Updated and revised with permission from Aunger et al. [1] under CC-BY 4.0 license

Our finalised typology is depicted in Fig. 3 in a Venn-style diagram. The position of a behaviour in the typology shows the distinct dimensions which are important to how it is defined. If a behaviour is located within an area of overlapping dimensions, this means that all these dimensions apply to the behaviour in question. As a worked example, we can look at the differences between bullying and harassment. Most definitions we identified for bullying stated that bullying: requires a hierarchy, is inherently frequent, highly targeted, and visible (hence its position in the typology within those overlapping circles). Harassment, in contrast, shares dimensions of inherent frequency, targetedness, and visibility with bullying, but does not require a hierarchy, and specifically targets an individual’s protected characteristics (as defined in UK law). Neither are necessarily physical in nature.

The typology also clarifies which behaviours are related to each other or are synonymous. For example, some more ‘passive’ - or ‘less-targeted’ - behaviours including negative workplace behaviour, disruptive behaviour, and deviant behaviour are used largely interchangeably in the academic literature and in practice. Non-targeted UBs within these types may include being repeatedly late for work, or procrastination, and these can reduce workplace effectiveness. Such passive behaviours may impact patient safety and staff wellbeing by reducing workplace efficiency - but may not work in the same way as targeted behaviours such as incivility and bullying (e.g. because targeted types can inhibit psychological safety) [5].

## Discussion

This new typology sets out key dimensions common across different types of staff-to-staff UB and clarifies important similarities and differences between them. This is important because misunderstandings can undermine intervention effectiveness by underestimating prevalence of UB, inhibiting speaking up, and enable instigators to avoid accountability. We found that measuring UBs depends strongly upon how questions are asked and what behaviours are mentioned in the process. Often, use of umbrella terms such as ‘unprofessional behaviour’ or using ‘bullying’ as a catch-all term can be insufficient for describing the range of behaviours affecting staff [4]. Hence, this typology depicts the range of UBs that healthcare organisations need to capture to properly assess the prevalence of UB.

We identified five dimensions inherent to UBs between staff, namely: visibility; inherent frequency; whether they are highly targeted; whether behaviours target protected characteristics such as race or sex, whether behaviours are physical; and if hierarchy is required. Some of these dimensions have implications for how they can be addressed; for example, others have explored the impact of hierarchy with UB types such as bullying, outlining how hierarchies can form across organisational as well as larger societal boundaries where knowledge, experience, and social support can influence interpersonal dynamics [30]. Our recent realist review of the drivers of UB identified hierarchy as a key means for making staff easier to target and inhibiting ability to speak up [3]. Similarly, addressing invisible behaviours may be substantially more challenging than those that are more evident to witnesses and recipients [31]. Staff from ethnic minority backgrounds are particularly affected by less visible UBs such as microaggressions such as comments on appearance or undermining [32] and, to date, interventions have done little to address this [16]. Lastly, while some behaviours are less targeted than others, such as general rudeness, evidence has shown that these behaviours are extremely widespread and still have significant impacts on patient safety and staff wellbeing [33].

### Comparison with other typologies

One other typology of UBs in healthcare has been developed. This typology was developed based on direct reports of UB drawn from a reporting system of types of unprofessional conduct at a large hospital in the USA [34]. These authors, rather than looking at common dimensions across behaviours, developed a typology using qualitative content analysis of these reports [34]. Due to also employing a wider focus that also considered staff-to-patient behaviours, they found four higher-order categories comprising: competent medical care, clear and respectful communication, integrity, and responsibility. They found that coworker reports most frequently describe disrespectful and offensive communication rather than other types of UB, findings supported by similar publications from a reporting system in Australia [35]. The authors report that this typology is being actively used to help triage messages in their reporting system intervention [36]. This demonstrates how such typologies can be of practical use to healthcare organisations.

Another older, but more widely used example originates with Robinson and Bennett (1995) [37]. They developed a typology of deviant workplace behaviours across sectors, drawing on a multidimensional scaling methodology involving three phases [37]. This typology set out behaviours into a quadrant with two main dimensions: organisational vs interpersonal, and minor vs. serious. While we did identify a similar dimension of targeted vs. less targeted, we did not attempt to situate behaviours in terms of seriousness due to the subjectivity inherent to one’s own experience. Within the quadrants were four categories of behaviour: production deviance - minor organisational (e.g., leaving early, taking excessive breaks); property deviance - serious organisational (e.g., sabotage, theft); political deviance - minor interpersonal (e.g., favouritism, gossiping); and personal aggression - serious interpersonal (e.g., harassment, verbal abuse). We focused predominately on interpersonal behaviour rather than production or property deviance behaviours outlined by Robinson and Bennett – however, it is important to note that such behaviours exist with real impact on organisations too. The typology by Robinson and Bennett (1995) may have broader utility for understanding impacts across a range of organisational types and has a stronger focus on organisational productivity, whereas the typology outlined by us may have more healthcare-specific utility for understanding impact on staff wellbeing and patient safety.

### Use in policy and practice

Typologies may have a range of uses in addressing staff behaviour. For example, to create a more uniform understanding of when staff have experienced UBs from co-workers, a first step is to clarify what is - and is not - considered unprofessional by members of the organisation, underpinned by organisational values and professional codes of conduct. Ideally, organisations should draw upon an evidence-based typology to inform code of conduct development, such as the typology outlined here, to ensure that the differences between behaviours (e.g. bullying versus incivility), are clear. Examples of what is and is not acceptable should be provided, capturing the range of behaviours set out in the typology. A behaviours and values statement or document should be highly visible and discussed with new employees during induction. To enhance its impact, staff can also be asked to sign a conduct pledge [31].

Organisations can also hold staff training and education sessions to ensure they are aware of these policies and what behaviours are and are not acceptable [16]. However, to foster psychological safety, these small steps should be accompanied by wider culture-change programmes [16]. At a national scale, policy change can also help. Currently, in the UK, only harassment and discrimination are protected against in law. Defining bullying (and other UBs if possible) in law in the UK would provide uniformity in understanding across organisations and would better-protect staff - not only in healthcare, but also elsewhere [38].

Improving consistency of understanding of UB is an important step towards addressing UB between staff and its impact on patient safety and staff psychological wellbeing, but much more needs to be done. Our recent review [16] and guide [39], produced for practitioners (free to download at https://www.workforceresearchsurrey.health), highlights a range of strategy options for interventions as well as recommendations for implementation. To advance research in this area, academics and practitioners can build upon the research and typology presented here, to foster a more consistent understanding of UBs. This can aid the development of theory-informed prevention interventions to minimise and prevent UB between healthcare staff, ultimately improving staff wellbeing at work and patient care quality and safety. Further research could test the typology via a Delphi process or other forms of empirical process to ensure its practical utility.

### Strengths and limitations

Strengths of this typology include its basis in a wide-ranging review of international literature that synthesised many definitions of UB types, and the use of stakeholder involvement at multiple timepoints to test and ‘sense-check’ the findings. However, one limitation is that this article is based on a realist review that sought to understand interventions to reduce UB, and therefore the review did not consider interventions to improve civility or professionalism. As such, this article and typology is focused on *un*professional behaviours only. Another limitation is that the typology of UBs was formulated using a structured process with stakeholder engagement but may still be subject to subjective disagreements about whether certain behaviours are, for example, visible or invisible. Use of a more robust consensus building methodology such as a Delphi process could have strengthened the evidence upon which the typology was based. Lastly, use of independent coding by more than one researcher could have improved the objectivity of the analysis.

## Conclusion

Inconsistent understandings of unprofessional behaviours could undermine implementation of interventions by inhibiting ability to measure unprofessional behaviour and understand where it is happening, inhibiting speaking up, and enabling instigators to avoid accountability. This inconsistency has also extended to the literature, such as in how specific terms such as bullying or harassment are defined. Our novel typology of these behaviours clarifies differences between them based on underlying dimensions of visibility, inherent frequency, whether they are highly targeted, if behaviours target protected characteristics, if they are physical in nature, and if hierarchy is required. We encourage future use of the typology by organisations, healthcare practitioners, intervention architects, and other academics, to clarify terms and move towards a more uniform understanding of unprofessional behaviours.

## Electronic supplementary material

Below is the link to the electronic supplementary material.

Supplementary Material 1

Supplementary Material 2

Supplementary Material 3

## Data Availability

The datasets used and/or analysed during the current study are available from the corresponding author on reasonable request.
