# Supplementary material for: Developing a novel typology of unprofessional behaviours between healthcare staff: a best fit framework synthesis
Source: BMC Health Serv Res. 2026 Jan 24;26:262. doi: 10.1186/s12913-025-13962-5 (PMC12911253; doi:10.1186/s12913-025-13962-5)
Supplement: Supplementary file 2 — Supplementary Material 2 [file 12913_2025_13962_MOESM2_ESM.docx]

# Full characteristics of included sources

| **Document** | **Source type / study design** | **Country of conduct or focus** | **Sample** | **Healthcare type** | **Area of unprofessional behaviour** | **Key findings & relevancy** |
| --- | --- | --- | --- | --- | --- | --- |
| Step 1 – Initial theories | | | | | | |
| Ariza-Montes et al. (2013)[1] | Survey | EU member states | Sub-sample of 284 health professionals | General healthcare | Bullying | Draws on data from the 5^th^ European Working Conditions Survey to identify predictors of bullying in the healthcare workplace. Predictors included shift working, monotonous tasks, stress, and few promotion opportunities. |
| Armstrong (2018)[2] | Systematic review | n/a | Nurses, 10 studies | General healthcare | Incivility | A systematic review of strategies used to address incivility in the nursing workplace. It found that some strategies such as communication training and education may have some promise for managing incivility. But studies were low quality. |
| Bambi et al. (2017)[3] | Systematic review | n/a | Nurses, 7 studies | General healthcare | Incivility, lateral violence, and bullying | Assesses strategies to prevent incivility between nurses. Limited number of studies were available, so it was not possible to assess effectiveness of existing programmes. More innovative interventions are needed. |
| Barzallo Salazar et al. (2014)[4] | Simulation study / RCT design | USA | 55 trainees, encouraged (n=28) and discouraged (n=27) groups | Surgery | ‘Discouraging environment’ | This study is RCT that explores how surgeon behaviour affects trainee willingness to speak up during surgeries. Positive surgeon behaviour leads to increased trainee speaking up and improved patient safety. |
| BBC News (2021)[5] | News article | UK | n/a | General healthcare | Bullying | News article reporting on a £3.4 million settlement to current and former staff at NHS Highland. |
| Benjamin (2021)[6] | Opinion article | UK | n/a | General healthcare | Microaggressions | Opinion article summarising what microaggressions are and how they are experienced in the healthcare workplace. Offers also some accounts from those who have experienced them. |
| Blackstock, Salami and Cummings (2018)[7] | Integrative review | n/a | Nurses, 22 studies | General healthcare | Horizontal violence | An integrative review exploring the organisational antecedents of horizontal violence among nurses. Working conditions, organisational culture, and leadership roles were all found to be key themes. |
| British Medical Association (2017)[8] | Narrative review | UK | Doctors, unclear number of studies | General healthcare | Bullying and harassment | A review of research by the British Medical Association assessing prevalence of bullying and harassment affecting doctors, as well as antecedents and solutions. |
| British Medical Association (2018)[9] | Report | UK | n/a | General healthcare | Bullying and harassment | Report by the British Medical Association about how to address bullying and harassment in the healthcare workplace including suggestions such as intervening early. |
| British Medical Association (2021)[10] | Web page | UK | n/a | General healthcare | Unacceptable behaviour | Web page defining what unacceptable behaviours are in the healthcare workplace, and setting out some informal resolution options. |
| Cooper (2018)[11] | Opinion piece | UK | n/a | General healthcare | Bullying | Highlights experience of bullying by different people in the NHS with quotes. Highlights some strategies people can take to address it, such as documenting incidences. |
| Cruz, Rodriguez and Mastropaolo (2019)[12] | Cross-sectional study | USA | 296 African American and Latino participants | General healthcare | Microaggressions | Study seeking to provide psychometric evidence for the Microaggressions in Health Care Scale. Provides significant background information regarding microaggressions. Microaggressions were found to correlate with mental health symptoms. |
| Felblinger (2009)[13] | Editorial paper | n/a | n/a | General healthcare | Bullying, incivility, and disruptive behaviours | An editorial that assesses how to identify bullying, incivility and disruptive behaviours, as well as how to mitigate their impact. Factors such as changing hierarchies, conflicting loyalties, and stress were stated to increase risk of such behaviour. |
| General Medical Council (2015)[14] | Report drawing on interviews | n/a | 12 sites | Surgery and obstetrics and gynaecology | Undermining and bullying | A report and review of bullying and undermining behaviours in medical education and training. Explores the groups that experience such behaviours more, factors contributing to them, and wider context for such behaviours. |
| Gillespie et al. (2017)[15] | Intervention | USA | Five academic campuses, nursing students | General healthcare | Bullying | Article describing the development process and utility of an educational intervention for use by nursing faculty with nursing students performed in a university context. |
| Illing et al. (2013)[16] | Evidence synthesis | n/a | 160 papers, 55 described in detail | General healthcare | Bullying and harassment | An evidence synthesis exploring occurrence, causes, and management of bullying and harassment behaviours in healthcare. |
| Illing et al. (2016)[17] | Report | UK | n/a | General healthcare | Bullying | A review of measures that are appropriate for measuring change in bullying over time in healthcare organisations. |
| Jones and Kelly (2014)[18] | Editorial paper | UK | n/a | General healthcare | Organisational deafness | An editorial exploring organisational deafness and highlights how people often do speak up but that their concerns fall on deaf ears. Suggests some means to attempt to resolve this. |
| Kaiser (2017)[19] | Survey | USA | 237 staff nurses | General healthcare | Incivility | Exploration of leadership style and the relationship to nurse-to-nurse incivility. Transformational leadership was found to correlate with lower levels of incivility. However, leadership style was not a very strong factor affecting incivility incidence. |
| Keller et al. (2020)[20] | Systematic review | n/a | 53 included papers | General healthcare | Incivility | Systematic review identifying predictors of incivility within healthcare teams. It found conceptualisation of incivility was subjective and varied, and quality of studies low. Results were inconsistent regarding individual characteristics but situational and cultural predictors were identified. |
| Kline (2021)[21] | Opinion piece / online blog | UK | n/a | General healthcare | Racism | Explores the risks associated with not tackling racism in the NHS. These include depriving patients of the best talent, blame culture affecting patient safety, impact to staff health, loss of patient benefit attained by greater representation. |
| Maben et al. (2012)[22] | Mixed methods case study | UK | 66 survey respondents, 18 staff interviews | Acute care | Incivility and bullying | Explores the link between staff experience of work and care received by acutely ill older people. Finds that incivility and bullying led to worsened patient care experience. |
| Mannion et al. (2019)[23] | Literature review and textual analysis | UK | Unclear number of documents, 7 in textual analysis | General healthcare | Misconduct | Examines how doctor misconduct can be understood using metaphors of bad apples, barrels, or orchards. |
| Mitchell (2021)[24] | Editorial | UK | n/a | Acute care | Bullying | An editorial exploring findings of bullying and racial discrimination at a NHS trust as a result of an inspection. |
| National Guardian’s Office (2018)[25] | Report | UK | Data from 7087 cases | General healthcare | Bullying and harassment | An overview of cases of speaking up to Freedom To Speak Up guardians in the NHS between Apr 2017 and Apr 2018. Shows that 45% of cases included an element of harassment and bullying. |
| Nursing & Midwifery Council (2021)[26] | Report | UK | n/a | General healthcare | Bullying | Report on the register of nurses from April 2020 to 2021 during the Covid-19 pandemic. Reports on instance of bullying in this time. |
| Parizad et al. (2018)[27] | Qualitative study | Iran | 15 registered nurses | Emergency department | Unprofessional behaviours | Explores Iranian nurse’s experiences of unprofessionalism in the emergency department. Explores contributors to these behaviours as well as prevalence. |
| Pislakov et al. (2013)[28] | Literature review | USA | Unclear number of sources | Healthcare in general | Bullying and aggressive behaviour | An informal literature review that explores what types of people engage in bullying, how it is defined, and its causes. |
| Quinlan et al. (2014)[29] | Scoping review | n/a | 8 sources | Healthcare in general | Bullying | A scoping review exploring interventions to reduce bullying in healthcare organisations. It revealed eight articles that included education, championing, and zero-tolerance policies. |
| Riskin et al. (2015)[30] | Simulation study RCT | USA | 24 neonatal intensive care unit teams | Neonatal intensive care unit | Rudeness | A simulation RCT with exposure to rudeness or control conditions. It found that rudeness had adverse consequences on information sharing and help-seeking behaviours with implications for patient safety. |
| Riskin et al. (2017)[31] | Simulation study RCT | USA | 39 neonatal intensive care unit teams | Neonatal intensive care unit | Rudeness | A simulation RCT with exposure to rudeness or control, as well as rudeness with and without a preventative or therapeutic intervention. It was found that cognitive bias modification was able to mitigate adverse effects of rudeness. |
| Rogers-Clark, Pearce and Cameron (2009)[32] | Systematic review | n/a | 24 papers | General healthcare | Disruptive behaviour | A systematic review of interventions to manage disruptive clinician behaviour in the nursing work environment. It found few sources evaluating effectiveness of interventions. |
| Ross et al. (2020)[33] | Report drawing on interviews | UK | 12 NHS staff and three case studies of NHS Providers | Acute care | Racism | A report investigating workforce race inequalities and inclusion in NHS providers. Assesses lived experience of a number of NHS staff as well as interventions used to address these race inequalities in NHS provider case studies. |
| Salin (2003)[34] | Review | n/a | Non-healthcare, bullying in general | Non-healthcare | Bullying | A review assessing ways of understanding workplace bullying in general and the factors that precipitate it. Such factors include antecedents including power imbalances, low perceived costs, and motivating structures such as reward systems. |
| Walton (2006)[35] | Editorial | n/a | n/a | Healthcare in general | Hierarchy and power | Explores the impact of hierarchy on the ability to speak up from a patient safety perspective. |
| Westbrook et al. (2018a)[36] | Editorial | Australia | n/a | Healthcare in general | Unprofessional behaviour | Highlights the impact of unprofessional behaviour in the Australian health care system, as well as the limited intervention evidence base while calling for greater culture change efforts. |
| Wild et al. (2015)[37] | Editorial | UK | n/a | Surgery | Undermining and bullying | Recommendations by the Association of Surgeons in Training to help create a positive learning environment free of undermining and bullying. |
| Yu et al. (2019)[38] | Systematic review with narrative synthesis | n/a | 38 articles | General healthcare | Resilience | This systematic review aimed to identify the personal and work-related factors associated with nurse resilience. The study concluded that understanding nurse resilience can help promote personal and professional growth and reduce the effects of job demands. |
| Step 2 – Exhaustive search | | | | | | |
| Academy of Medical Royal Colleges (2016)[39] | Report | UK | n/a | Trainees (general) | Undermining and bullying | Explores causes of UB based on findings of a one-day seminar and proposes strategies |
| Al-Ghabeesh and Qattom (2019)[40] | Survey | Jordan | 120 ED nurses | Emergency department | Bullying | Explores quantitatively the antecedents of bullying in a sample of ED nurses and proposes in limited form some measures to mitigate it |
| Al-Rias (2017)[41] | Editorial | UK | n/a | Emergency department | ‘Handover hostility’ | Describes a scenario of handover hostility in the English NHS and explores reasons for it |
| Allen (2015)[42] | Narrative review | UK | n/a | General healthcare | Bullying | Narrative review of bullying with a focus on the English NHS. Presents causes of bullying and preventative measures one can take. |
| Almost et al. (2010)[43] | Testing theoretical model | Canada | 277 acute care nurses | Acute care | Conflict | Quantitatively testing a theoretical model of causes of intra-group conflict among nurses with a non-experimental design. Explores how factors like self-evaluation, complexity of nursing care, and interactional justice affect incidence of conflict. |
| Alspach (2007)[44] | Editorial | No specific country | n/a | General healthcare | Lateral hostility | Explores causes, impacts, and prevention of lateral hostility between critical care nurses. |
| Anderson (2011)[45] | Editorial | Australia | n/a | Emergency department | Workplace aggression & horizontal violence | Explores violence from patients but also horizontal violence, its causes, the harm it causes, and how legislation may reduce it. |
| Anonymous (2018)[46] | Case study | No specific country | n/a | Acute care | Bullying | Exploration of how a single event of bullying in the operating room led to an impact on patient safety. |
| Armstrong (2017)[47] | Intervention | USA | 9 nurses | Acute care | Incivility | Quantitative investigation of an intervention in a rural hospital to reduce nursing workplace incivility. Findings indicated no change in experience of incivility but a greater ability to respond to incivility. |
| Asi Karakaş and Okanli (2015)[48] | Intervention | Turkey | 30 nurses | Acute care | Mobbing | This study reported an evaluation of an assertiveness training intervention in 30 nurses who experienced a high level of mobbing. Results indicated a statistically significant fall in mobbing after the intervention and an increase in assertiveness. |
| Babenko-Mould and Laschinger (2014)[49] | Survey | Canada | 126 year 4 nursing students | Acute care | Incivility | Focuses on effect on incivility on nurse burnout and talks about strategies to mitigate this impact. It finds that incivility is strongly related to burnout. |
| Babla et al. (2021)[50] | Letter | UK | n/a | Critical care | Microaggressions | Focuses on racial microaggressions and what they are, how they manifest in the healthcare workplace, and how they should be addressed. |
| Barrett et al. (2009)[51] | Intervention | USA | 59 pre-intervention and 45 post-intervention nurses | Critical care | Lateral violence | The study assessed a team-building intervention to reduce lateral violence, using mixed methods. The intervention was found to improve group cohesion. |
| Beale and Leather (2005)[52] | Report | UK | n/a | General healthcare | ‘Working relationships’ | Guide by the Royal College of Nursing to help improve collegiality and avoid unprofessional behaviours in the nursing workplace. Presents team and individual assessment tools to improve team and individual behaviours. Also explores what behaviour may or may not be bullying. |
| Blackstock et al. (2022)[53] | Review | n/a | 15 resources | General healthcare | Incivility | A review which develops an ecological model to understanding co-worker incivility experiences of new graduate nurses. |
| Blakey et al. (2018)[54] | Editorial | Australia | n/a | Trainees (general) | Bullying | Explores in in-depth manner the reasons why bullying interventions may and may not be effective, or even counterproductive, with trainees. |
| Carter et al. (2013)[55] | Mixed methods survey + interview | UK | Seven NHS trusts in NE England, comprising 2950 NHS staff and 43 in qualitative telephone interview | General healthcare (acute, primary and mental) | Bullying | Survey with qualitative elements also which explores prevalence and impact of bullying in the NHS. Largely focuses on prevalence but also has rich description from qualitative findings of impacts of bullying. They find bullying is prevalent in the NHS with 20% of staff reporting being bullied. |
| Ceravolo et al. (2012)[56] | Intervention | USA | 4032 practicing nurses, 1160 students and faculty | Acute care | Lateral violence | This intervention used culture-change and communication enhancing workshops to decrease lateral violence in a five-hospital integrated health and care system. |
| Chadwick and Travaglia (2017)[57] | Systematic Review | Australia | 62 studies | General healthcare | Bullying | Explores what types of behaviour comprise bullying, the contributing factors, and factors that can help address it too. |
| Chipps and McRury (2012)[58] | Intervention | USA | 16 staff members | Rehabilitation | Bullying | This quasi-experimental study assessed pre-test and post-test an educational intervention to address workplace bullying. They noted an increase in bullying reports as a result (from <1 act weekly/daily to 1.6 acts weekly/daily), but it did help develop a learning community. |
| Churchman and Doherty (2010)[59] | Qualitative interviews | UK | 12 nurses | Acute care | ‘Challenging doctors’ practice’ | Unpacks when nurses would be willing to challenge doctors’ practice without fear of reprisal or conflict and the interprofessional status of nurses vs. doctors. |
| Churruca et al. (2022)[60] | Intervention | Australia | Eight hospitals | Acute care | Respect | Reports on a case study of the Ethos messaging system implemented across eight hospitals in Australia. |
| Clark, Ahten and Macy (2013)[61] | Intervention | USA | 25 nurses on two units | General healthcare | Lateral violence | Study of an intervention which used PBL to address incivility. It was found to heighten awareness of incivility, improve favourable reaction to PBL, and give increased confidence. |
| Colangelo (2019)[62] | Editorial | n/a | n/a | Acute care | Bullying | Reports on the ETHOS programme and how some staff feel that the intervention is unfairly applied. |
| Credland and Whitfield (2022)[63] | Qualitative study | UK | 14 interviews | Paramedics | Incivility | A qualitative study of the experience of paramedics with incivility in the UK. It highlights the impact on clinical decision making and wellbeing. |
| Dahlby and Herrick (2014)[64] | Intervention | USA | 29 nurses pretest, 29 posttest | Acute care | Lateral violence | A study examining the effects of a 1.5 hour educational intervention on lateral violence. |
| Demarco, Roberts, and Chandler (2005)[65] | Intervention | USA | 5 graduate nursing students | Acute care | Group cohesion | Pilot study investigating a writing group’s ability to build group cohesion. The group writing drew on the ”Amherst Writers and Artists” method which did not focus on UB but nonetheless was intended to help cope with it. |
| Dimarino (2011)[66] | Intervention | USA | Unclear | Ambulatory setting | Lateral violence | Reporting of one ambulatory surgery centre’s approach to reducing lateral violence through education about lateral violence, and zero tolerance policies. Did not test effectiveness. |
| Dixon-Woods et al. (2019)[67] | Intervention | USA | 67 employees (20 senior, 47 frontline) | Acute care | Disruptive behaviour | This study sought to improve employee’s ability to speak up about transgressive and disruptive behaviour at a John Hopkins Medicine hospital. |
| Edwards and O’Connell (2007)[68] | Narrative review | UK | n/a | Nurse education | Bullying | Delves deeply into bullying and its aetiology and presents recommendations for practice to help tackle it. |
| Efe and Ayaz (2010)[69] | Mixed methods survey + focus groups | Turkey | 206 nurses in one hospital and four focus groups with 16 total participants | Acute care | Mobbing | Primarily investigated the prevalence of mobbing but also presents rich qualitative data regarding the causes of mobbing and what might be done about it. Suggests assertiveness training and need to solve communication issues. |
| Embree, Bruner and White (2013)[70] | Intervention | USA | 143 nurses | Acute care | Lateral violence | Investigates effectiveness of a cognitive rehearsal education intervention on nurse to nurse lateral violence. |
| Gamble Blakey et al. (2019)[71] | Narrative review | No specific country | 38 articles | General healthcare | Bullying | Explores interventions to help students with bullying and explores catalysts for bullying, how policies may affect bullying, how targeting of specific groups affects interventions, framing to improve effectiveness, and skills of facilitators. |
| Griffin (2004)[72] | Intervention | USA | 26 newly licensed nurses | Acute care | Lateral violence | Reporting an intervention assessing use of cognitive rehearsal techniques to reduce lateral violence between newly licensed nurses. |
| Griffith et al. (2019)[73] | Intervention | USA | 25 clinical staff | Emergency department | Mistreatment | The authors developed an educational advance programme to aid residents and faculty in understanding and improving their learning environment. Attendees proposed coaching, signage, zero tolerance policies, and more, to tackle mistreatment. |
| Hawkins, Jeong and Smith (2019)[74] | Integrative review | No specific country | Sixteen papers included | Acute care | Negative workplace behaviour | Delves into the conceptual differences in terms dealing with negative workplace behaviour, precipitating factors, and interventions to reduce such behaviour. |
| Hemmings et al. (2021)[75] | Report | UK | n/a | General healthcare | Non-inclusive culture | Report by NHS Employers and the Nuffield trust focused on means of attracting and keeping a diverse NHS workforce. Explores a number of case studies in rich detail as well as interventions used to improve diversity and why they may have failed. |
| Hickson et al. (2007([76] | Intervention | USA | Unknown | Acute care | Unprofessional behaviours | Outlines and reviews the Vanderbilt approach to identifying, measuring, and addressing UB using four graduated interventions. |
| Hughes (2003)[77] | Editorial | UK | n/a | Acute care | Bullying | Looks at strategies at both an individual and organisational level to reduce bullying and its impact in healthcare. |
| Hutchinson et al. (2008)[78] | Outlining theoretical model | No specific country | n/a | General healthcare | Bullying | Investigates a novel model of bullying in the nursing workplace which includes organisational antecedents and consequences of such behaviour. Goes into detail about the organisational climate that enables bullying. |
| Hutchinson et al. (2010)[79] | Testing theoretical model | Australia | 370 nurses | General healthcare | Bullying | Confirms the strengths of the relationships outlined in the model above – as such, explores several causes of bullying and how these may work. |
| Işik et al. (2020)[80] | Qualitative interviews | Turkey | Two hospitals with 14 interviews with perioperative nurses | Acute care | Communication failure | Qualitatively investigates reasons for communication failures in perioperative environment, and as part of this looks at intra-team violence and other causes of communicative failures that might lead to negative behaviour. |
| Jenkins et al. (2011)[81] | Intervention | USA | Unknown | General healthcare | Civility | Explores how 6 monthly hour-long journal club meetings may increase civility. Also incorporated journalling. |
| Johnson and May (2015)[82] | Systematic review | No specific country | 67 articles | General healthcare | Professionalism | A systematic overview of systematic reviews using normalisation process theory to interpret the results. It found that interventions using normative restructuring of practice, modifying peer group norms, and emphasising expectations of external groups, had the most success. |
| Johnson et al. (2020)[83] | Simulation | USA | 58 students | General healthcare (students) | Incivility | An RCT simulation investigating the impact of an occurrence of incivility on clinical performance, teamwork and emotions. It found that 66% of the experimental group made a major error in their cardiopulmonary resuscitation performance. |
| Kang and Jeong (2019)[84] | Intervention | South Korea | 72 hospital nurses | Acute care | Bullying | Investigates a cognitive rehearsal smartphone-based intervention’s impact on bullying using a cluster quasi-randomised trial. |
| Kang, Kim and Yun (2017)[85] | Intervention | South Korea | 40 nurses | Acute care | Bullying | An RCT to investigate a cognitive rehearsal programme on workplace bullying. |
| Kile et al. (2019)[86] | Intervention | USA | 19 nurses | Community | Incivility | Investigated with a mixed methods pilot study the impact of incivility and cognitive rehearsal education on nurse-to-nurse incivility. |
| Kline (2022)[87] | Case study | UK | 34 interviews and survey of 3506 people at one NHS Trust | Acute care | Bullying | A review of bullying and harassing behaviours at one NHS Trust, using mixed methods. It found significant evidence of negative culture at the Trust and proposes several strategies to implement to tackle this. |
| Lasater et al. (2015)[88] | Intervention | USA | 94 nursing staff | Acute care | Incivility | Study of a three-part educational intervention to see if it reduced incivility in two units of a hospital. |
| Laschinger et al. (2012)[89] | Intervention | Canada | 8 units with 33 controls | Acute care | Civility | Investigates the impact of the CREW intervention over 6 months with 8 intervention units and 33 control units. |
| Leiter et al. (2011)[90] | Intervention | Canada | 1,173 workers across 41 units | Acute care | Civility | Assesses the effect of 6 months of the CREW intervention and found that greater group x time interactions were found in the intervention group for civility, supervisor incivility, respect, cynicism, job satisfaction, and management trust, and absences. |
| Lewis (2018)[91] | Report (mixed methods) | UK | 110+ employees interviewed, 1488+ surveyed (1100 full responses) | Emergency department | Unprofessional behaviours | Report on grievances about staff behaviour at an NHS Ambulance Trust. Explores unreasonable management behaviours and their causes, incivility and disrespect, rudeness, and threatening behaviour. Delves deeply into the qualitative data. |
| Longo and Hain (2014)[92] | Editorial | No specific country | n/a | Nephrology | Bullying | With a focus on dialysis centres, talks about healthy work environments that can counteract inappropriate work behaviours, including collaboration, effective decisionmaking, authentic leadership, etc. |
| Lovejoy-Bluem (2016)[93] | Editorial | No specific country | n/a | Neonatal | Incivility | Discusses incivility in the neonatal ICU, and spends most of its words on discussing strategies to reduce incivility. Also walks through an example of how small incivility can lead to large clinical impact. |
| Manton (2017)[94] | Editorial | No specific country | n/a | Emergency | Bullying | An editorial exploring bullying, its effect on the work environment, and strategies to reduce it. |
| Markwell et al. (2015)[95] | Editorial | Australia | n/a | Students (general healthcare) | Performance management vs. bullying and harassment | Breaks down performance management and how in some cases it may be considered bullying while in other cases it might not be. Presents briefly some strategies to tackle this issue. Also presents a couple ‘vignettes’ as illustrative examples. |
| McKenzie et al. (2019)[96] | Intervention | Australia | 21 healthcare staff pre-to-post | Acute care | Unprofessional behaviour | Investigated factors affecting implementation of a multistrategy intervention using education, reporting systems with graduated intervention processes, safety champions, and action plans, to tackle unprofessional behaviour. |
| Mello and Jagsi (2020)[97] | Editorial | No specific country | n/a | General healthcare | Sexual harassment | Delves into the details of how to respond to sexual harassment, but mostly in an informal manner. |
| Miller and Chen (2021)[98] | Editorial | No specific country | n/a | Students (general healthcare) | Microaggressions | Tackles strategies to address microaggressions at a programmatic and institutional level, as well as informally as an individual. |
| National Freedom to Speak Up Guardian (2018)[99] | Report | UK | n/a | General healthcare | Bullying | Presents some examples of how various NHS trusts have sought to tackle bullying at their organisations. |
| Nicotera, Mahon and Wright (2014)[100] | Intervention | USA | 36 working nurses | Acute care | Conflict | Assesses a nursing conflict intervention using mixed pre and post-test methods with a control sample, which sought to reduce structurational divergence by raising awareness of it and teaching skills to manage it. |
| NHS Employers (2016)[101] | Report | UK | n/a | Paramedics | Bullying | Explores many real-world strategies implemented by NHS ambulance trusts and how these strategies have been received. |
| Nikstatis and Simko (2014)[102] | Intervention | USA | 21 nurses | Acute care | Incivility | A quantitative pilot study using a 1-group pre and post intervention test design to assess a 60 minute educational programme. |
| O’Connell, Garbark and Nader (2019)[103] | Intervention | USA | 76 participants | Military | Lateral violence | A quantitative exploration of nurses’ perceptions of lateral violence within a military setting and the impact of an education, cognitive rehearsal, and role play intervention. |
| O’Keefe, Brennan and Doherty (2022)[104] | Description of an intervention | Ireland | 203 course participants | General healthcare | Conflict | Describes an intervention, an instructional course, designed to improve professionalism, and how it is implemented. Course content focuses on conflict management. |
| Osatuke et al. (2009)[105] | Intervention | USA | 647 post-intervention CREW participants and 680 comparison (total 34 workgroups) | Acute care | Civility | Preliminary evaluation of a nationwide Veterans Health Administration interventions called CREW across 23 sites. |
| Owens, Singh and Cribb (2019)[106] | Editorial | UK | n/a | General healthcare | Professionalism | Explores the impact of austerity on professional working environments in healthcare in the UK context. Suggests mechanisms for how austerity signals to workers and organisations the degree to which professionalism is valued. |
| Pavithra et al. (2022)[107] | Qualitative study | Australia | 1636 survey respondents (various healthcare staff) | Acute care | Unprofessional behaviour | Explores qualitative responses to two open-ended survey questions delivered to seven hospitals in Australia. It found that a perceived lack of organisational action erodes confidence in hospital leaders and ability to address and mitigate UB effectively. |
| Parker et al. (2016)[108] | Intervention | USA | Unclear / organisation-wide | Acute care | Horizontal violence | Explores how nurses at an acute care hospital were able to implement multiple interventions to reduce horizontal violence prevalence in the organisation. These included 13 total organisational, leadership, and individual level strategies. |
| Phillips et al. (2018)[109] | Integrative review | No specific country | 38 studies | General healthcare | Incivility | Explores quite deeply the different antecedents for incivility in healthcare. It also delves into preventative strategies and leadership behaviours, education, and systems thinking. |
| Purpora and Blegen (2012)[110] | Theoretical model | No specific country | n/a | General healthcare | Horizontal violence | Describes a theoretical model of how horizontal violence comes to impact the quality and safety of patient care. Model includes oppression and explores the concept in relation to oppression, as well as how this leads to internalisation and affects communication etc. |
| Rocker (2008)[111] | Narrative review | Canada | n/a | General healthcare | Bullying | A narrative review which explores strategies to prevent nurse to nurse bullying, mitigate its effects, legal responses in Canada, and its origins. |
| Royal College of Nursing (2014)[112] | Report | UK | n/a | General healthcare | Bullying and harassment | Guidance materials from the RCN oriented towards organisations for preventing bullying and harassment in healthcare. Provides information about the law, the impact of bullying, prevention and reactive strategies and sample policies and recommendations for investigating incidents. |
| Royal College of Surgeons of England (2021)[113] | Report | UK | n/a | Acute care | Disruptive behaviour | Guide from the RCSE for surgeons delving deeply into how to tackle disruptive behaviour, as well as what causes it. |
| Rutherford, Gillespie and Smith (2019)[114] | Integrative review | No specific country | 22 articles | Trainees (general healthcare) | Bullying | Integrative review which focuses on interventions against bullying of prelicensure students specifically. Explores policy-level interventions, as well as those at individual or organisational levels. |
| Saxton (2012)[115] | Intervention | USA | 17 perioperative nurses | Surgery | Disruptive behaviour | Development and evaluation of a communication skills intervention to improve perceived self-effectiveness of perioperative nurses using a pre-post design. |
| Sheehan, McCabe and Garavan (2020)[116] | Testing a theoretical model | Ireland | 1507 employees from 47 hospitals | Acute care | Bullying | A study testing a moderated mediated model of workplace bullying and employee outcomes. Explores the mediating influence of well-implemented anti-bullying HR practices and what well-implemented looks like. Delves into factors which comprise good implementation in the model. |
| Shuttleworth (2018)[117] | News article | UK | n/a | Emergency department | Bullying | News article highlighting how an NHS trust has tried to tackle its culture of bullying after a whistleblower came forward. |
| Sillero and Buil (2021)[118] | Qualitative interviews | Spain | 16 healthcare professionals | Acute care | Interprofessional collaboration | Examines the interactions between doctors and nurses and how they can be kept civil. Hence it sheds light on how interprofessional collaboration as a cause can lead to incivility. |
| Solheim (2018)[119] | Editorial | No specific country | n/a | Emergency department | Lateral violence | Discusses lateral violence in nursing and how it is much too prevalent. Mentions some informal means to tackle poor behaviour. |
| Speck et al. (2014)[120] | Intervention | USA | Three hospitals | Acute care | Unprofessional behaviour | Assessment of a professionalism committee approach to tackling unprofessional behaviour across three large teaching hospitals. |
| Stagg et al. (2017)[121] | Intervention | USA | 10 nurses | Acute care | Bullying | Assessed the effectiveness of a 2-hour cognitive rehearsal programme, 6 months after completion. |
| Stagg et al. (2011)[122] | Intervention | USA | 20 nurses | Acute care | Bullying | Evaluated a workplace bullying cognitive rehearsal programme. |
| Stevens (2002)[123] | Intervention | USA | Unclear | Acute care | Bullying | Informally explores the impact of a multi-strategy anti-bullying intervention in a large teaching hospital, delivered mostly through workshops for education, policies, supervisor training, and more. |
| Stone, Philips and Douglas (2019)[124] | Qualitative interviews | Australia | Six female doctors who were in training when abused | General healthcare | Sexual harassment and assault | Discusses in depth the assaults that happened to several female doctors in the workplace by other doctors and how they tried to address what happened. |
| Tame (2012)[125] | Qualitative interviews | UK | 23 perioperative nurses at one NHS Trust | Acute care | Horizontal violence | Qualitative study investigating experience of horizontal violence at an NHS Trust and the causes for it, the personal impacts, and touches upon strategies to resolve it. |
| Taylor and Taylor (2018)[126] | Editorial | No specific country | n/a | General healthcare | Horizontal violence | A discussion paper based on a prior review and experience of the authors which argues that horizontal violence is a quality improvement concern. As part of this it explores strategies such as codes of conduct and how these should be implemented across different contexts. |
| Thorsness and Sayors (1995)[127] | Intervention | USA | Approximately 100 surgical staff | Acute care | Conduct issues | Evaluation of a programme adopting a systems approach to cultivating a positive work environment for perioperative staff members |
| Tran (2015)[128] | Editorial | Australasia | n/a | Emergency department | Bullying and harassment | A practical guide for how trainees should deal with bullying and harassment. Presents a flowchart for decision-making for how to approach the situation in the Australasian context with legal routes if necessary. |
| Tuffour (2022)[129] | Qualitative interviews | UK | Five nurses from sub-Saharan Africa | Mental health | Discrimination | Goes into depth on the experience of nurses from Africa working in the UK NHS Context. As part of this it explores their experience of discrimination and marginalisation and the ‘snowy peak’ syndrome. Paper makes recommendations for how to decrease discrimination in the NHS. |
| Venkatesh et al. (2016)[130] | Communication | Australasia | Unclear | Acute care | Bullying, discrimination, and sexual assault | Response of College of Intensive Care Medicine of Australia and New Zealand to survey showing high prevalence of bullying, discrimination and sexual assault in their workplace. Outlines actions and longer-term strategies they will be implementing in response. |
| Villafranca et al. (2017)[131] | Narrative review | No specific country | n/a | Acute care | Disruptive behaviour | Explores the antecedents of disruptive behaviour at intrapersonal, organisational, and interpersonal levels. It also discusses the prevention and management options to tackle it. |
| Warrner et al. (2016)[132] | Intervention | USA | 60-bed orthopaedic inpatient unit incl. management | Acute care | Incivility | Evaluates an intervention comprising awareness education, cognitive rehearsal, and which included management. |
| Weaver (2013)[133] | Editorial | No specific country | n/a | General healthcare (graduates) | Horizontal violence and bullying | Tackles the chain of violence in nursing from one generation to another and seeks to understand how to reduce it from individual, school, and organisational levels. |
| Webb et al. (2016)[134] | Intervention | USA | Three hospitals | Acute care | Disrespectful and unsafe behaviours | Presents a feasibility study of the Co-Worker Observation Reporting System implemented by Vanderbilt University Medical Centre to reduce disrespectful and unsafe behaviours. |
| Wild et al. (2015)[37] | Editorial | UK | n/a | Acute healthcare (trainees) | Undermining and bullying | Editorial which looks at the definitions of undermining and bullying, implications of such behaviours towards trainees, and how such behaviours can be tackled. Towards the end it presents specific recommendations. |
| Wilson (2016)[135] | Narrative review | No specific country | n/a | General healthcare | Bullying | Narrative review that explores the root causes, types of perpetrators, behaviours of bullying, and interventions to combat it. |
| Zhang and Xiong (2019)[136] | Review | No specific country | 44 articles | General healthcare | Horizontal violence | Review focused on the impact of nursing and coping strategies that help tackle the effects of bullying as well as reduce it. These include educational interventions, leadership styles, and enlightenment. |
| Step 2 – Search Update | | | | | | |
| Adams and Bryan (2021)[137] | Editorial | Canada | n/a | General healthcare | Harassment | Examines the role of leadership as the ones in a strategic position to address harassment and bullying. |
| Baldwin et al (2022)[138] | Intervention | USA | Three academic medical centres | Acute care | Professionalism | Descriptive study analysing the types of reports received during the intervention to promote professionalism with nurses. |
| Bamberger and Bamberger (2022)[139] | Editorial | n/a | n/a | General healthcare | Unacceptable behaviours | Editorial exploring the impact of unacceptable behaviours between healthcare workers on patient safety. |
| Banerjee et al. (2022)[140] | Intervention | USA | Division faculty members (n = 41) and pulmonary and critical care fellows (n = 12) | Acute care | Racism | Assessed the feasibility of a year-long antiracism educational study. As it was mostly a feasibility study, postintervention surveying indicated a 15% increase in self-directed learning on related topics. |
| Bry and Wigert (2022)[141] | Qualitative study | Sweden | 13 neonatal nurses | Neonatal intensive care | Organisational climate and interpersonal interactions | Explores the organisational climate and type of interpersonal interactions experienced by registered nurses in the neonatal unit. As part of this it explores the impact of incivility. |
| Hawkins et al. (2022b)[142] | Intervention | Australia | 230 nurses from 12 units in four hospitals | Acute care | Negative workplace behaviour | Examined experiences of negative workplace behaviour and ways of coping with nursing staff before and after educational workshops. They did not find statistically significant results. |
| Hawkins et al. (2022a)[143] | Qualitative study | Australia | 13 nurses | Acute care | Negative workplace behaviour | A qualitative study exploring negative workplace behaviour with nurses and why it occurs. It finds that while some individuals can be more inclined than others to do so, it is facilitated by organisational influences. |
| Kousha et al. (2022)[144] | Intervention | Iran | 80 emergency nurses | Emergency care | Incivility | Examines the effectiveness of an educational and cognitive rehearsal programme among emergency nurses. |
| Naylor, Boyes and Killingback (2022)[145] | Qualitative study | UK | 6 physiotherapists | Acute care | Incivility | Investigates the impact of incivility on physiotherapists working in the acute hospital setting using Interpretative Phenomenological Analysis. |
| Westbrook et al. (2013)[146] | Intervention | Australia | Staff across five hospitals | Acute care | Unprofessional behaviours | Investigates changes in prevalence of unprofessional behaviours following a professional accountability programme called Ethos being implemented. |

1. Ariza-Montes A, Muniz NM, Montero-Simó MJ, Araque-Padilla RA. Workplace bullying among healthcare workers. Int J Environ Res Public Health. 2013;10(8):3121–39.

2. Armstrong N. Management of Nursing Workplace Incivility in the Health Care Settings: A Systematic Review. Work Heal Saf. 2018;66(8):403–10.

3. Bambi S, Guazzini A, de Felippis C, Lucchini A, Rasero L. Preventing workplace incivility, lateral violence and bullying between nurses. A narrative literature review. Acta Biomed. 2017;88(12):39–47.

4. Barzallo Salazar MJ, Minkoff H, Bayya J, Gillett B, Onoriode H, Weedon J, et al. Influence of surgeon behavior on trainee willingness to speak up: A randomized controlled trial. J Am Coll Surg [Internet]. 2014;219(5):1001–7. Available from: http://dx.doi.org/10.1016/j.jamcollsurg.2014.07.933

5. BBC News. NHS Highland pays out millions to bullied staff [Internet]. 2021. p. 1–6. Available from: https://www.bbc.co.uk/news/uk-scotland-highlands-islands-58718290

6. Benjamin A. Names, hair, identity and micro aggressions. British Medical Association [Internet]. 2021;(1):1–5. Available from: https://www.bma.org.uk/news-and-opinion/names-hair-identity-and-micro-aggressions

7. Blackstock S, Salami B, Cummings GG. Organisational antecedents, policy and horizontal violence among nurses: An integrative review. J Nurs Manag. 2018;26(8):972–91.

8. British Medical Association. Workplace bullying and harassment of doctors A review of recent research [Internet]. British Medical Association. 2017. Available from: file:///Users/VWC/Downloads/Bullying and harassment research review v7 WEB.pdf

9. British Medical Association. Bullying and harassment : how to address it and create a supportive and inclusive culture. 2018.

10. British Medical Association. Advice and support. 2021 [cited 2021 Oct 20]. p. 1–8 Promoting a positive working environment. Available from: https://www.bma.org.uk/advice-and-support/discrimination-and-harassment/bullying-and-harassment/promoting-a-positive-working-environment

11. Cooper K. BMA. 2018. Ending the silence. Available from: https://www.bma.org.uk/news-and-opinion/ending-the-silence

12. Cruz D, Rodriguez Y, Mastropaolo C. Perceived microaggressions in health care: A measurement study. PLoS One. 2019;14(2):1–11.

13. Felblinger DM. Bullying, incivility, and disruptive behaviors in the healthcare setting: identification, impact, and intervention. Front Health Serv Manage. 2009;25(4):13–23.

14. General Medical Council. Building a supportive environment: a review to tackle undermining and bullying in medical education and training. General Medical Council. 2015.

15. Gillespie GL, Grubb PL, Brown K, Boesch MC, Ulrich DL. “Nurses eat their young”: A novel bullying educational program for student nurses. J Nurs Educ Pract. 2017;7(7):11.

16. Illing J, Carter M, Thompson NJ, Crampton PES, Morrow GM, Howse JH, et al. Evidence synthesis on the occurrence, causes, management of bullying and harassing behaviours to inform decision making in the NHS. Final report. Vol. 44, NIHR Service Delivery and Organisation Programme. 2013.

17. Illing J, Thompson N, Crampton P, Charlotte M, Ms R, Kehoe A, et al. Workplace bullying: measurements and metrics to use in the NHS Final Report for NHS Employers [Internet]. 2016. Available from: http://www.nhsemployers.org/~/media/Employers/Documents/Campaigns/NHS_Employers_Bullying_Measures_Final_Report.pdf

18. Jones A, Kelly D. Deafening silence? Time to reconsider whether organisations are silent or deaf when things go wrong. BMJ Qual Saf. 2014;23(9):709–13.

19. Kaiser JA. The relationship between leadership style and nurse-to-nurse incivility: turning the lens inward. J Nurs Manag. 2017;25(2):110–8.

20. Keller S, Yule S, Zagarese V, Parker SH. Predictors and triggers of incivility within healthcare teams: A systematic review of the literature. BMJ Open. 2020;10(6):1–15.

21. Kline R. Middlesex University London. 2021. Racism which impacts healthcare staff endangers patient care. Available from: https://mdxminds.com/2021/11/22/racism-which-impacts-healthcare-staff-endangers-patient-care/

22. Maben J, Adams M, Peccei R, Murrells T, Robert G. “Poppets and parcels”: The links between staff experience of work and acutely ill older peoples’ experience of hospital care. Int J Older People Nurs. 2012;7(2):83–94.

23. Mannion R, Davies H, Powell M, Blenkinsopp J, Millar R, McHale J, et al. Healthcare scandals and the failings of doctors: Do official inquiries hold the profession to account? J Health Organ Manag. 2019;33(2):221–40.

24. Mitchell G. Bullying and inadequate leadership found at flagship nursing trust. Nursing Times. 2021;1–6.

25. National Guardian’s Office. Speaking up in the NHS in England: A summary of speaking up to Freedom to Speak Up Guardians in NHS trusts and foundation trusts. 2018.

26. Nursing & Midwifery Council. The NMC register. 2021.

27. Parizad N, Hassankhani H, Rahmani A, Mohammadi E, Lopez V, Cleary M. Nurses’ experiences of unprofessional behaviors in the emergency department: A qualitative study. Nurs Heal Sci. 2018;20(1):54–9.

28. Pisklakov S, Tilak V, Patel A, Xiong M. Bullying and Aggressive Behavior among Health Care Providers: Literature Review. Adv Anthropol. 2013;03(04):179–82.

29. Quinlan E, Robertson S, Miller N, Robertson-Boersma D. Interventions to reduce bullying in health care organizations: A scoping review. Heal Serv Manag Res. 2014;27(1):33–44.

30. Riskin A, Erez A, Foulk TA, Kugelman A, Gover A, Shoris I, et al. The impact of rudeness on medical team performance: A randomized trial. Pediatrics. 2015;136(3):487–95.

31. Riskin A, Erez A, Foulk TA, Riskin-Geuz KS, Ziv A, Sela R, et al. Rudeness and medical team performance. Pediatrics. 2017;139(2):1–11.

32. Rogers-Clark C, Pearce S, Cameron M. Management of disruptive behaviour within nursing work environments: a comprehensive systematic review of the evidence. JBI Libr Syst Rev. 2009;7(15):615–78.

33. Ross S, Jabbal J, Chauhan K, Maguire D, Randhawa M, Dahir S. Workforce race inequalities and inclusion in NHS providers. 2020;(July).

34. Salin D. Ways of explaining workplace bullying: A review of enabling, motivating and precipitating structures and processes in the work environment. Hum Relations. 2003;56(10):1213–32.

35. Walton MM. Hierarchies: The Berlin wall of patient safety. Qual Saf Heal Care. 2006;15(4):229–30.

36. Westbrook J, Sunderland N, Atkinson V, Jones C, Braithwaite J. Endemic unprofessional behaviour in health care: the mandate for a change in approach. Med J Aust [Internet]. 2018;209(9):380–1. Available from: https://doi.org/10.5694/mja17.01261

37. Wild JRL, Ferguson HJM, McDermott FD, Hornby ST, Gokani VJ. Undermining and bullying in surgical training: A review and recommendations by the Association of Surgeons in Training. Int J Surg. 2015;23:S5–9.

38. Yu F, Raphael D, Mackay L, Smith M, King A. Personal and work-related factors associated with nurse resilience: A systematic review. Int J Nurs Stud [Internet]. 2019;93:129–40. Available from: https://doi.org/10.1016/j.ijnurstu.2019.02.014

39. Academy of Medical Royal Colleges. Creating supportive environments: Tackling behaviours that undermine a culture of safety [Internet]. AoMRC Trainee Doctors’ Group. Academy of Medical Royal Colleges; 2016. Available from: http://www.aomrc.org.uk/wp-content/uploads/2016/09/Creating_supportive_environments_280916-2.pdf

40. Al-Ghabeesh SH, Qattom H. Workplace bullying and its preventive measures and productivity among emergency department nurses. BMC Health Serv Res [Internet]. 2019;19(1):44. Available from: https://ovidsp.ovid.com/ovidweb.cgi?T=JS&CSC=Y&NEWS=N&PAGE=fulltext&D=pmnm4&AN=31101071

41. Al-Rais A. Why we should avoid handover hostility. BMJ [Internet]. 2017;356:j1272. Available from: https://ovidsp.ovid.com/ovidweb.cgi?T=JS&CSC=Y&NEWS=N&PAGE=fulltext&D=med14&AN=28320693

42. Allen B. Understanding bullying in healthcare organisations. Nurs Stand. 2015;30(14):50–60.

43. Almost J, Doran DM, Mcgillis Hall L, Spence Laschinger HK. Antecedents and consequences of intra-group conflict among nurses. J Nurs Manag. 2010;18(8):981–92.

44. Alspach G. Critical care nurses as coworkers: are our interactions nice or nasty? Crit Care Nurse. 2007;27(3):10–4.

45. Anderson K. Workplace aggression and violence: nurses and midwives say NO. Aust Nurs J. 2011;19(1):26–9.

46. Anonymous. When bullying affects patient safety. AORN J. 2018;108(1):78–80.

47. Armstrong NE. A Quality Improvement Project Measuring the Effect of an Evidence-Based Civility Training Program on Nursing Workplace Incivility in a Rural Hospital Using Quantitative Methods. Online J Rural Nurs Heal Care [Internet]. 2017;17(1):100–37. Available from: https://search.ebscohost.com/login.aspx?direct=true&db=cin20&AN=123121492&site=ehost-live

48. Asi Karakaş S, Okanli AE. The Effect of Assertiveness Training on the Mobbing That Nurses Experience. Work Heal Saf. 2015;63(10):446–51.

49. Babenko-Mould Y, Laschinger HKS. Effects of incivility in clinical practice settings on nursing student burnout. Int J Nurs Educ Scholarsh [Internet]. 2014;11(1):145–54. Available from: https://ovidsp.ovid.com/ovidweb.cgi?T=JS&CSC=Y&NEWS=N&PAGE=fulltext&D=med11&AN=25367690

50. Babla K, Lau S, Akindolie O, Radia T, Modi N, Kingdon C, et al. Racial microaggressions within respiratory and critical care medicine. Lancet Respir Med [Internet]. 2021;9(3):e27–8. Available from: http://dx.doi.org/10.1016/S2213-2600(21)00001-1

51. Barrett A, Piatek C, Korber S, Padula C. Lessons learned from a lateral violence and team-building intervention. Nurs Adm Q. 2009;33(4):342–51.

52. Beale D, Leather P. Working with care – improving working relationships in health and social care: self-assessment tools for health and social care teams [Internet]. Healthy workplace, healthy you. Dignity at work. Royal College of Nursing - RCN; 2005. Available from: https://www.rcn.org.uk/-/media/royal-college-of-nursing/documents/publications/2015/september/004972.pdf?la=en

53. Blackstock S, Cummings G, Glanfield F, Yonge O. A review: Developing an ecological model approach to co‐worker incivility experiences of new graduate nurses. J Adv Nurs. 2022;(April 2021):1–16.

54. Blakey AG, Anderson L, Smith-Han K, Wilkinson T, Collins E, Berryman E. Time to stop making things worse: An imperative focus for healthcare student bullying research. N Z Med J. 2018;131(1479):81–5.

55. Carter M, Thompson N, Crampton P, Morrow G, Burford B, Gray C, et al. Workplace bullying in the UK NHS: A questionnaire and interview study on prevalence, impact and barriers to reporting. BMJ Open. 2013;3(6):1–12.

56. Ceravolo DJ, Schwartz DG, Foltz-Ramos KM, Castner J. Strengthening communication to overcome lateral violence. J Nurs Manag. 2012;20(5):599–606.

57. Chadwick S, Travaglia J. Workplace bullying in the Australian health context: a systematic review. J Heal Organ Manag. 2017;31(3):286–301.

58. Chipps EM, McRury M. The development of an educational intervention to address workplace bullying: A pilot study. J Nurses Staff Dev. 2012;28(3):94–8.

59. Churchman JJ, Doherty C. Nurses’ views on challenging doctors’ practice in an acute hospital. Nurs Stand. 2010;24(40):42–7.

60. Churruca K, Pavithra A, McMullan R, Urwin R, Tippett S, Cunningham N, et al. Creating a culture of safety and respect through professional accountability: case study of the Ethos program across eight Australian hospitals. Aust Heal Rev. 2022;46(3):319–24.

61. Clark CM, Ahten SM, Macy R. Using Problem-Based Learning Scenarios to Prepare Nursing Students to Address Incivility. Clin Simul Nurs [Internet]. 2013;9(3):e75–83. Available from: http://dx.doi.org/10.1016/j.ecns.2011.10.003

62. Colangelo A. St Vincent’s reviews anti-bullying program amid staff backlash. The Age [Internet]. 2019;1–3. Available from: https://www.theage.com.au/national/victoria/st-vincent-s-reviews-anti-bullying-program-amid-staff-backlash-20190223-p50zri.html

63. Credland NJ, Whitfield C. Incidence and impact of incivility in paramedicine: A qualitative study. Emerg Med J [Internet]. 2022;39(1):52–6. Available from: https://ovidsp.ovid.com/ovidweb.cgi?T=JS&CSC=Y&NEWS=N&PAGE=fulltext&D=medl&AN=34039640

64. Dahlby MA, Herrick LM. Evaluating an educational intervention on lateral violence. J Contin Educ Nurs. 2014;45(8):344–50.

65. Demarco RF, Roberts SJ, Chandler GE. The Use of a Writing Group to Enhance Voice and Connection Among Staff Nurses. J Nurses Prof Dev. 2005;21(3):85–90.

66. Dimarino TJ. Eliminating Lateral Violence in the Ambulatory Setting: One Center’s Strategies. AORN J [Internet]. 2011;93(5):583–8. Available from: https://ovidsp.ovid.com/ovidweb.cgi?T=JS&CSC=Y&NEWS=N&PAGE=fulltext&D=med8&AN=21530706

67. Dixon-Woods M, Campbell A, Martin G, Willars J, Tarrant C, Aveling EL, et al. Improving Employee Voice about Transgressive or Disruptive Behavior: A Case Study. Acad Med. 2019;94(4):579–85.

68. Edwards SL, O’Connell CF. Exploring bullying: Implications for nurse educators. Nurse Educ Pract [Internet]. 2007;7(1):26–35. Available from: https://www.sciencedirect.com/science/article/pii/S1471595306000485

69. Efe SY, Ayaz S. Mobbing against nurses in the workplace in Turkey. Int Nurs Rev [Internet]. 2010;57(3):328–34. Available from: https://ovidsp.ovid.com/ovidweb.cgi?T=JS&CSC=Y&NEWS=N&PAGE=fulltext&D=med8&AN=20796062

70. Embree JL, Bruner DA, White A. Raising the Level of Awareness of Nurse-to-Nurse Lateral Violence in a Critical Access Hospital. Nurs Res Pract. 2013;2013:1–7.

71. Gamble Blakey A, Smith-Han K, Anderson L, Collins E, Berryman E, Wilkinson TJ. Interventions addressing student bullying in the clinical workplace: A narrative review. BMC Med Educ. 2019;19(1):1–13.

72. Griffin M. Teaching cognitive rehearsal as a shield for lateral violence: an intervention for newly licensed nurses. J Contin Educ Nurs. 2004;35(6):257–63.

73. Griffith M, Clery MJ, Humbert B, Joyce JM, Perry M, Hemphill RR, et al. Exploring Action Items to Address Resident Mistreatment through an Educational Workshop. West J Emerg Med. 2019;21(1):42–6.

74. Hawkins N, Jeong S, Smith T. New graduate registered nurses’ exposure to negative workplace behaviour in the acute care setting: An integrative review. Int J Nurs Stud [Internet]. 2019;93:41–54. Available from: https://doi.org/10.1016/j.ijnurstu.2018.09.020

75. Hemmings N, Buckingham H, Oung C, Palmer W. Attracting, supporting and retaining a diverse NHS workforce [Internet]. 2021. Available from: www.nuffieldtrust.org.uk/research

76. Hickson GB, Pichert JW, Webb LE, Gabbe SG. A complementary approach to promoting professionalism: Identifying, measuring, and addressing unprofessional behaviors. Acad Med. 2007;82(11):1040–8.

77. Hughes A. Being bullied what an insight. Br J Perioper Nurs. 2003;13(4):166–72.

78. Hutchinson M, Jackson D, Wilkes L, Vickers MH. A new model of bullying in the nursing workplace organizational characteristics as critical antecedents. Adv Nurs Sci. 2008;31(2):60–71.

79. Hutchinson M, Wilkes L, Jackson D, Vickers MH. Integrating individual, work group and organizational factors: Testing a multidimensional model of bullying in the nursing workplace. J Nurs Manag [Internet]. 2010;18(2):173–81. Available from: https://www.ncbi.nlm.nih.gov/pubmed/20465745

80. Işık I, Gümüşkaya O, Şen S, Arslan Özkan H. The Elephant in the Room: Nurses’ Views of Communication Failure and Recommendations for Improvement in Perioperative Care. AORN J. 2020;111(1):e1–15.

81. Jenkins S, Woith W, Kerber C, Stenger D. Why can’t we all just get along? A civility journal club intervention. Nurse Educ. 2011;36(4):140–1.

82. Johnson MJ, May CR. Promoting professional behaviour change in healthcare: What interventions work, and why? A theory-led overview of systematic reviews. BMJ Open. 2015;5(9).

83. Johnson SL, Haerling KA, Yuwen W, Huynh V, Le C. Incivility and Clinical Performance, Teamwork, and Emotions: A Randomized Controlled Trial. J Nurs Care Qual. 2020;35(1):70–6.

84. Kang J, Jeong YJ. Effects of a smartphone application for cognitive rehearsal intervention on workplace bullying and turnover intention among nurses. Int J Nurs Pract. 2019;25(6):1–10.

85. Kang J, Kim JI, Yun S. Effects of a cognitive rehearsal program on interpersonal relationships, workplace bullying, symptom experience, and turnover intention among nurses: A randomized controlled trial. J Korean Acad Nurs. 2017;47(5):689–99.

86. Kile D, Eaton M, DeValpine M, Gilbert R. The effectiveness of education and cognitive rehearsal in managing nurse-to-nurse incivility: A pilot study. J Nurs Manag. 2019;27(3):543–52.

87. Kline R. A review into culture and bullying at University Hospitals of North Midlands NHS Trust. 2022;(March).

88. Lasater K, Mood L, Buchwach D, Dieckmann NF. Reducing incivility in the workplace: Results of a three-part educational intervention. J Contin Educ Nurs. 2015;46(1):15–24.

89. Laschinger HKS, Leiter MP, Day A, Gilin-Oore D, MacKinnon SP. Building empowering work environments that foster civility and organizational trust: Testing an intervention. Nurs Res. 2012;61(5):316–25.

90. Leiter MP, Laschinger HKS, Day A, Oore DG. The impact of civility interventions on employee social behavior, distress, and attitudes. J Appl Psychol. 2011;96(6):1258–74.

91. Lewis D. Workplace Culture at Southwestern Ambulance NHS Foundation Trust. 2018.

92. Longo J, Hain D. Bullying: a hidden threat to patient safety. Nephrol Nurs J. 2014;41(2):193–9; quiz 200.

93. Lovejoy-Bluem A. Incivility and/or Human Kind(ness) in the NICU. Acad Neonatal Nurs. 2016;35(3):173–4.

94. Manton AP. Bullying: A Pebble in the Pond. J Emerg Nurs [Internet]. 2017;43(5):389–90. Available from: https://ovidsp.ovid.com/ovidweb.cgi?T=JS&CSC=Y&NEWS=N&PAGE=fulltext&D=med14&AN=28822462

95. Markwell A, Smith S, Michalski M, Conroy S, Bell A. Performance management versus bullying and harassment: An educator perspective. EMA - Emerg Med Australas [Internet]. 2015;27(5):468–72. Available from: https://search.ebscohost.com/login.aspx?direct=true&db=cin20&AN=109542016&site=ehost-live

96. McKenzie LN, Shaw L, Jordan JE, Alexander M, O’Brien M, Singer SJ, et al. Factors Influencing the Implementation of a Hospitalwide Intervention to Promote Professionalism and Build a Safety Culture: A Qualitative Study. Jt Comm J Qual Patient Saf. 2019;45(10):694–705.

97. Mello MM, Jagsi R. Standing Up against Gender Bias and Harassment — A Matter of Professional Ethics. N Engl J Med [Internet]. 2020;6(1):510–2. Available from: nejm.org

98. Miller DT, Chen EH. Helping the learner to deal with microaggressions in the workplace: Individual, programmatic, and institutional-level responses. AEM Educ Train [Internet]. 2021;5(S1):S140–3. Available from: https://ovidsp.ovid.com/ovidweb.cgi?T=JS&CSC=Y&NEWS=N&PAGE=fulltext&D=pmnm5&AN=34616989

99. National Freedom to Speak Up Guardian. Bullying behaviour is unacceptable. It is unprofessional and unneccessary. It affects the wellbeing of individuals and the teams within which they work. 2018.

100. Nicotera AM, Mahon MM, Wright KB. Communication that builds teams: Assessing a nursing conflict intervention. Nurs Adm Q. 2014;38(3):248–60.

101. NHS Employers. Tackling bullying in ambulance trusts: a guide for action. 2016;

102. Nikstaitis T, Simko LC. Incivility among intensive care nurses: The effects of an educational intervention. Dimens Crit Care Nurs [Internet]. 2014;33(5):293–301. Available from: https://ovidsp.ovid.com/ovidweb.cgi?T=JS&CSC=Y&NEWS=N&PAGE=fulltext&D=med11&AN=25140748

103. O’Connell KM, Garbark RL, Nader KC. Cognitive Rehearsal Training to Prevent Lateral Violence in a Military Medical Facility. J Perianesthesia Nurs. 2019;34(3):645-653.e1.

104. O’Keeffe DA, Brennan SR, Doherty EM. Resident Training for Successful Professional Interactions. J Surg Educ [Internet]. 2022;79(1):107–11. Available from: https://doi.org/10.1016/j.jsurg.2021.08.017

105. Osatuke K, Moore SC, Ward C, Dyrenforth SR, Belton L. Civility, Respect, Engagement in the Workforce (CREW). J Appl Behav Sci. 2009;45(3):384–410.

106. Owens J, Singh G, Cribb A. Austerity and Professionalism: Being a Good Healthcare Professional in Bad Conditions. Heal Care Anal [Internet]. 2019;27(3):157–70. Available from: https://search.ebscohost.com/login.aspx?direct=true&db=cin20&AN=137793764&site=ehost-live

107. Pavithra A, Sunderland N, Callen J, Westbrook J. Unprofessional behaviours experienced by hospital staff: qualitative analysis of narrative comments in a longitudinal survey across seven hospitals in Australia. BMC Health Serv Res [Internet]. 2022;1–15. Available from: https://doi.org/10.1186/s12913-022-07763-3

108. Parker KM, Harrington A, Smith CM, Sellers KF, Millenbach L. Creating a Nurse-Led Culture to Minimize Horizontal Violence in the Acute Care Setting: A Multi-Interventional Approach. J Nurses Prof Dev. 2016;32(2):56–63.

109. Phillips JM, Stalter AM, Winegardner S, Wiggs C, Jauch A. Systems thinking and incivility in nursing practice: An integrative review. Nurs Forum [Internet]. 2018;53(3):286–98. Available from: https://www.ncbi.nlm.nih.gov/pubmed/29359482

110. Purpora C, Blegen MA. Horizontal Violence and the Quality and Safety of Patient Care: A Conceptual Model. Nurs Res Pract. 2012;2012(May 2012):1–5.

111. Rocker CF. Addressing nurse-to-nurse bullying to promote nurse retention. Online J Issues Nurs. 2008;13(3):1–10.

112. Royal College of Nursing. Bullying and harassment: good practice guidance for preventing and addressing bullying and harassment in health and social care organisations. Vol. 66. 2014.

113. Royal College of Surgeons of England. MANAGING DISRUPTIVE BEHAVIOURS IN SURGERY: A Guide to Good Practice [Internet]. 2021. Available from: www.rcseng.ac.uk/standardsandguidance

114. Rutherford DE, Gillespie GL, Smith CR. Interventions against bullying of prelicensure students and nursing professionals: An integrative review. Nurs Forum. 2019;54(1):84–90.

115. Saxton R. Communication Skills Training to Address Disruptive Physician Behavior. AORN J [Internet]. 2012;95(5):602–11. Available from: https://ovidsp.ovid.com/ovidweb.cgi?T=JS&CSC=Y&NEWS=N&PAGE=fulltext&D=med9&AN=22541771

116. Sheehan M, McCabe TJ, Garavan TN. Workplace bullying and employee outcomes: a moderated mediated model. Int J Hum Resour Manag [Internet]. 2020;31(11):1379–416. Available from: https://www.tandfonline.com/doi/abs/10.1080/09585192.2017.1406390

117. Shuttleworth A. Can Arrowe Park show other trusts how to beat the bullies? Nurs Times [Internet]. 2018;114(3):12. Available from: https://ahs.idm.oclc.org/login?url=https://search.ebscohost.com/login.aspx?direct=true&db=ccm&AN=129664910&site=ehost-live&scope=site

118. Sillero AS, Buil N. Enhancing interprofessional collaboration in perioperative setting from the qualitative perspectives of physicians and nurses. Int J Environ Res Public Health [Internet]. 2021;18(20):1–11. Available from: https://www.mdpi.com/1660-4601/18/20/10775/pdf

119. Solheim J. Caring for Each Other While We Care for Others. J Emerg Nurs. 2018;44(4):319–20.

120. Speck RM, Foster JJ, Mulhern VA, Burke S V., Sullivan PG, Fleisher LA. Development of a professionalism committee approach to address unprofessional medical staff behavior at an academic medical center. Jt Comm J Qual Patient Saf. 2014;40(4):161–7.

121. Stagg SJ, Sheridan DJ, Jones RA, Speroni KG. Workplace Bullying: The Effectiveness of a Workplace Program. Aust Nurs midwifery J. 2017;24(9):34–6.

122. Stagg SJ, Sheridan D, Jones RA, Speroni KG. Evaluation of a Workplace Bullying Cognitive Rehearsal Program in a Hospital Setting. J Contin Educ Nurs. 2011;42(9):395–403.

123. Stevens S. Nursing workforce retention: Challenging a bullying culture. Health Aff. 2002;21(5):189–93.

124. Stone L, Phillips C, Douglas KA. Sexual assault and harassment of doctors, by doctors: a qualitative study. Med Educ [Internet]. 2019;53(8):833–43. Available from: https://onlinelibrary.wiley.com/doi/abs/10.1111/medu.13912

125. Tame S. The relationship between continuing professional education and horizontal violence in perioperative practice. J Perioper Pract. 2012;22(7):220–5.

126. Taylor RA, Taylor SS. Reframing and addressing horizontal violence as a workplace quality improvement concern. Nurs Forum. 2018;53(4):459–65.

127. Thorsness R, Sayers B. Systems Approach to Resolving Conduct Issues Among Staff Members. AORN J [Internet]. 1995;61(1):197–202. Available from: https://search.ebscohost.com/login.aspx?direct=true&db=cin20&AN=107404234&site=ehost-live

128. Tran V. Dealing with bullying and harassment: A practical guide for Australasian emergency medicine trainees. EMA - Emerg Med Australas. 2015;27(5):473–7.

129. Tuffour I. It is like ‘judging a book by its cover’: An exploration of the lived experiences of Black African mental health nurses in England. Nurs Inq. 2022;29(1):1–12.

130. Venkatesh B, Corke C, Raper R, Pinder M, Stephens D, Joynt G, et al. Findings of the bullying, discrimination and sexual harassment survey: Response of the college of intensive care medicine. Crit Care Resusc. 2016;18(4):228–9.

131. Villafranca A, Hamlin C, Enns S, Jacobsohn E. Disruptive behaviour in the perioperative setting: a contemporary review. Can J Anesth. 2017;64(2):128–40.

132. Warrner J, Sommers K, Zappa M, Thornlow DK. Decreasing work place incivility. Nurs Manage. 2016;47(1):22–30.

133. Weaver KB. The effects of horizontal violence and bullying on new nurse retention. J Nurses Prof Dev. 2013;29(3):138–42.

134. Webb LE, Dmochowski RR, Moore IN, Pichert JW, Catron TF, Troyer M, et al. Using coworker observations to promote accountability for disrespectful and unsafe behaviors by physicians and advanced practice professionals. Jt Comm J Qual Patient Saf. 2016;42(4):149–61.

135. Wilson JL. An exploration of bullying behaviours in nursing: a review of the literature. Br J Nurs. 2016;25(6):303–6.

136. Zhang X, Xiong L. Impact of Nurse Horizontal Violence and Coping Strategies: A Review. Yangtze Med. 2019;03(04):289–300.

137. Adams L, Bryan V. Workplace harassment: The leadership factor. Healthc Manag Forum [Internet]. 2021;34:81–6. Available from: https://journals.sagepub.com/home/HMF

138. Baldwin CA, Hanrahan K, Edmonds SW, Krumm AM, Sy A, Jones A, et al. Implementation of Peer Messengers to Deliver Feedback: An Observational Study to Promote Professionalism in Nursing. Jt Comm J Qual Patient Saf. 2022;000(i):1–12.

139. Bamberger E, Bamberger P. Unacceptable behaviours between healthcare workers: just the tip of the patient safety iceberg. BMJ Qual Saf [Internet]. 2022;31(9):638–41. Available from: https://www.ncbi.nlm.nih.gov/pubmed/35428683

140. Banerjee D, Nassikas NJ, Singh P, Andrea SB, Zhang AY, Aswad Y, et al. Feasibility of an Antiracism Curriculum in an Academic Pulmonary, Critical Care, and Sleep Medicine Division. Ats Sch. 2022;3(3):433–48.

141. Bry A, Wigert H. Organizational climate and interpersonal interactions among registered nurses in a neonatal intensive care unit: A qualitative study. J Nurs Manag (John Wiley Sons, Inc) [Internet]. 2022;30(6):2031–8. Available from: https://search.ebscohost.com/login.aspx?direct=true&db=cin20&AN=159455054&site=ehost-live

142. Hawkins N, Jeong SYS, Smith T, Sim J. Creating respectful workplaces for nurses in regional acute care settings: A quasi-experimental design. Nurs Open. 2022;(April 2022):78–89.

143. Hawkins N, Jeong SYS, Smith T, Sim J. A conflicted tribe under pressure: A qualitative study of negative workplace behaviour in nursing. J Adv Nurs. 2022;17(September):17.

144. Kousha S, Shahrami A, Forouzanfar MM, Sanaie N, Atashzadeh-Shoorideh F, Skerrett V. Effectiveness of educational intervention and cognitive rehearsal on perceived incivility among emergency nurses: a randomized controlled trial. BMC Nurs. 2022;21(1):153.

145. Naylor MJ, Boyes C, Killingback C. “You’ve broken the patient”: Physiotherapists’ lived experience of incivility within the healthcare team - An Interpretative Phenomenological Analysis. Physiotherapy [Internet]. 2022;117:89–96. Available from: https://doi.org/10.1016/j.physio.2022.09.001

146. Westbrook JI, Urwin R, McMullan R, Badgery-Parker T, Pavithra A, Churruca K, et al. Changes in the prevalence of unprofessional behaviours by co-workers following a professional accountability culture change program across five Australian hospitals. 2023.
