# Supplementary material for: Developing a novel typology of unprofessional behaviours between healthcare staff: a best fit framework synthesis
Source: BMC Health Serv Res. 2026 Jan 24;26:262. doi: 10.1186/s12913-025-13962-5 (PMC12911253; doi:10.1186/s12913-025-13962-5)
Supplement: Supplementary file 3 — Supplementary Material 3 [file 12913_2025_13962_MOESM3_ESM.docx]

This additional file outlines our mapping of definitions identified in the literature for various unprofessional behaviour related terms.

Table 1. Definitions of UB-related terms and underlying behaviours.

| **UB Types identified and coded according to definitions or features** | **Example definitions (where available) or behaviours** | **Number of definitions coded (if 0, then behaviours were not explicitly defined in the included literature)** |
| --- | --- | --- |
| Behaviours explicitly defined in the literature | | |
| Bullying | “Bullying is defined by Rodwell and Demir (2012) as a situation that occurs over a period of time where individuals perceive themselves to experience negative actions and behaviours from others. This can be carried out by one individual or several and the person being bullied has difficulty defending himself or herself from the abuse being experienced.” [1]  “Bullying is the misuse of power or position that undermines a person’s ability, or leaves them feeling hurt, frightened, angry or powerless.” [2]  “Bullying is behaviour that hurts or frightens someone who is less powerful, often forcing them to do something they do not want to do’” [3]  “repeated exposure to person, work and intimidation‐related negative acts such as abuse, teasing, ridicule, and social exclusion over a period of time in the workplace” [4] | 31 |
| Incivility | “low-intensity, deviant behaviors that are intended to harm the victim and demonstrate a lack of mutual respect.” [5]  “repeated offensive, abusive, intimidating, or insulting behavior, abuse of power, or unfair sanctions that make recipients upset and feel humiliated, vulnerable, or threatened, creating stress and undermining their self-confidence.” [6]  “rude or disruptive behaviors that violate workplace norms” [7] | 14 |
| Horizontal violence | “When behaviors are displayed among workers in the same rank, such as staff nurses, rather than across power gradients, it is referred to as horizontal or lateral violence” [8]  “a variety of unkind, discourteous, antagonistic interactions that occur between persons at the same organizational hierarchy level and are commonly described as divisive, infighting, backbiting, and off-putting” [9]  “Horizontal violence is behavior that is directed by one peer toward another that harms, disrespects, and devalues the worth of the recipient while denying them their basic human rights” [10] | 9 |
| Harassment | Legal UK definition: “unwanted conduct related to a relevant protected characteristic, which has the purpose or effect of violating an individual’s dignity or creating of intimidating, hostile, degrading, humiliating or offensive environment for that individual” [2]  “improper conduct by an individual, that is directed at and offensive to another individual in the workplace, including any event/location related to work, and that the individual knew or ought reasonably to have known would cause offence or harm.” [11] | 8 |
| Lateral violence | “any repetitive behavior among peers that is considered offensive, abusive, or intimidating by the target.” [12] | 6 |
| Disruptive behaviour | Behaviour that “tends to cause distress among other staff and affect overall morale within the work environment, undermining productivity and possibly leading to high staff turnover or even resulting in ineffective or substandard care.” [13]  “we deﬁne disruptive behaviour as constituting the following three criteria: a) interpersonal (i.e., directed toward others or occurs in the presence of others); b) results in a perceived threat to victims and/or witnesses; c) violates a reasonable person’s standard of respectful behaviour, as deﬁned in the Universal Declaration of Human Rights” [14] | 5 |
| Microaggressions | “Racial microaggressions are actions, words, or behaviours that undermine or insult a person’s character, abilities, or perceptions based on their ethnicity. Racial microaggressions have been classified into three major types: microassaults, microinsults, and microinvalidations” [15]  “stunning and automatic acts of disrespect arising from unconscious attitudes inflicted by the culturally dominant groups” [16] | 3 |
| Unprofessional behaviours | “Unprofessional behaviour encompasses a spectrum, from overtly hostile, bullying and inappropriate behaviours such as physical and verbal abuse, to more subtle behaviours such as lack of responsiveness, passive aggression, rudeness and incivility” [17]  “behavior that undermines a culture of safety” [18] | 3 |
| Mobbing | “Mobbing is the activity of a person to force someone out of the workplace through rumour, innuendo, intimidation, humiliation, discrediting and isolation” [19]  “unethical communication and antagonistic action directed by one or several people  toward a single individual systematically” [20] | 3 |
| Verbal abuse | “Communication perceived by a person to be a harsh, condemnatory attack, either professional or personal. Language intended to cause distress to a target” [9] | 3 |
| Negative workplace behaviour | “repeated, offensive, abusive, intimidating or insulting behaviour, abuse of power, or unfair sanctions that make recipients upset and feel humiliated, vulnerable, or threatened, creating stress and undermining their self-conﬁdence” [21] | 2 |
| Undermining | “Undermining is conduct that subverts, weakens or wears away a person's conﬁdence, and may occur when one practitioner intentionally or unintentionally erodes another practitioner's reputation or intentionally seeks to turn others against them” [22] | 2 |
| Violence | “the intentional use of physical force or power, threatened or actual, against oneself, another person, or against a group or community that either results in or has a high likelihood of resulting in injury, death, psychological harm, mal-development or deprivation” [23] | 2 |
| Mistreatment | “when behaviour shows disrespect for the dignity of others and unreasonably interferes with the learning process.” [24,25] | 2 |
| Sexual harassment | ‘any improper and unwelcome conduct that might reasonably be expected or be perceived to cause offence or humiliation to another person’ [26] | 1 |
| Gender bias | gender bias (manifested in discriminatory behavior that, though not necessarily consciously recognized by the perpetrator, is sexist) |  |
| Offensive displays | Displays of offensive material (e.g. flags) [27] | 1 |
| Sexual assault and violence | “an act of a sexual nature carried out against a person’s will or without their consent, through the use of physical force, intimidation or coercion and/or involving physical contact” [26] | 1 |
| Unethical behaviours | “negative or exclusionary interpretation and enactment of organisational values that are at odds with patient or staff identity and dignity” [28] | 1 |
| Victimisation | “Victimisation: is the unfair treatment of an employee as a direct consequence of raising or supporting a complaint of bullying and harassment.” [29] | 1 |
| Behaviours discussed but not defined in the literature | | |
| Minimisation | “having accomplishments and contributions disregarded” [30] | 0 |
| Humiliation | Being “publicly ridiculed (15%, n = 3), observed and followed (15%, n = 3), disgraced in front of coworkers (10%, n = 2), secluded by work organization (10%, n = 2), and intimidated (5%, n = 1)” [30] | 0 |
| Physical abuse | “Unwanted physical contact, Explicit physical threats or attacks, Suggestive gestures (such as mimicking the effects of a disability), Unnecessary touching or assault, Stalking which occurs at work or outside of work, but is related to work” [27] | 0 |
| Racism | “its definition including colourism, anti-Blackness in the Asian communities, and more recently, anti-Chinese sentiments in the wake of the COVID-19 pandemic” [15] | 0 |
| Aggression | “throwing things, hitting, slapping, breaking things, slamming doors, pushing, intimidating, stalking” [28] | 0 |
| Disrespect | “Participants reported different ways this ‘disrespect’ was expressed, both verbally and non- verbally. You’ll get a sort of derogatory look, you can see their eyes roll when you walk in with a patient, a bit of a tut and a bit of huffing and puffing” [31] | 0 |
| Ostracising | “’The consultant has given me strange looks in the department and tried not to make eye contact with me for over three months. This made me feel uncomfortable in the department and led to me avoiding them.’” [3] | 0 |
| Rudeness | “When discussing an example of rudeness from a colleague you feel belittled and embarrassed…you feel worthless, you feel completely worthless.” [31] | 0 |
| Scapegoating | “’Bully: “Have you noticed that whenever there is a code, Andy is usually working?’” [30] | 0 |
| Sexism | “Sexism was also used against women to give unfair workloads compared to men and to be used as mechanisms of control” [32] | 0 |
| Intimidation | “Conduct that belittles in some way, such as being shouted at, Intrusion by pestering, spying, following, Unnecessary closeness, Apportioning blame wrongly” [27] | 0 |
| Unacceptable behaviours | “using patronising and demeaning language; • shouting or talking aggressively to people; • unacceptable demeanour in meetings – actively looking disengaged and dismissive; • being overly critical of work, often in front of other colleagues; • not being listened to; • being rude and abrupt when direct reports ask questions: some participants talked about managers rolling their eyes when they asked a question” [33] | 0 |
| Unreasonable management | “‘Having your views and opinions ignored’, ‘Being given unmanageable workloads or impossible deadlines’, ‘Someone withholding information which affects your performance’, ‘Your employer not following proper procedures’, ‘Someone continually checking up on you or your work when it is not necessary’” [32] | 0 |

# References

1. Academy of Medical Royal Colleges. Creating supportive environments: Tackling behaviours that undermine a culture of safety [Internet]. AoMRC Trainee Doctors’ Group. Academy of Medical Royal Colleges; 2016 p. 1–19. Available from: <http://www.aomrc.org.uk/wp-content/uploads/2016/09/Creating_supportive_environments_280916-2.pdf>

2. Adams L, Bryan V. Workplace harassment: The leadership factor. Healthcare Management Forum. 2021;34:81–6.

3. Al-Ghabeesh SH, Qattom H. Workplace bullying and its preventive measures and productivity among emergency department nurses. BMC Health Services Research. 2019;19:44–44.

4. Al-Rais A. Why we should avoid handover hostility. BMJ (Online). 2017;356:j1272–j1272.

5. Allen B. Understanding bullying in healthcare organisations. Nursing Standard. 2015;30:50–60.

6. Almost J, Doran DM, Mcgillis Hall L, Spence Laschinger HK. Antecedents and consequences of intra-group conflict among nurses. Journal of Nursing Management. 2010;18:981–92.

7. Alspach G. Critical care nurses as coworkers: are our interactions nice or nasty? Critical Care Nurse. 2007;27:10–4.

8. Anderson K. Workplace aggression and violence: nurses and midwives say NO. Australian nursing journal (July 1993). 2011;19:26–9.

9. Anonymous. When bullying affects patient safety. AORN Journal. 2018;108:78–80.

10. Ariza-Montes A, Muniz NM, Montero-Simó MJ, Araque-Padilla RA. Workplace bullying among healthcare workers. International Journal of Environmental Research and Public Health. 2013;10:3121–39.

11. Armstrong N. Management of Nursing Workplace Incivility in the Health Care Settings: A Systematic Review. Workplace Health and Safety. 2018;66:403–10.

12. Armstrong NE. A Quality Improvement Project Measuring the Effect of an Evidence-Based Civility Training Program on Nursing Workplace Incivility in a Rural Hospital Using Quantitative Methods. Online Journal of Rural Nursing and Health Care. 2017;17:100–37.

13. Asi Karakaş S, Okanli AE. The Effect of Assertiveness Training on the Mobbing That Nurses Experience. Workplace Health and Safety. 2015;63:446–51.

14. Babenko-Mould Y, Laschinger HKS. Effects of incivility in clinical practice settings on nursing student burnout. International Journal of Nursing Education Scholarship. 2014;11:145–54.

15. Babla K, Lau S, Akindolie O, Radia T, Modi N, Kingdon C, et al. Racial microaggressions within respiratory and critical care medicine. The Lancet Respiratory Medicine. 2021;9:e27–8.

16. Baldwin CA, Hanrahan K, Edmonds SW, Krumm AM, Sy A, Jones A, et al. Implementation of Peer Messengers to Deliver Feedback: An Observational Study to Promote Professionalism in Nursing. The Joint Commission Journal on Quality and Patient Safety. 2022;000:1–12.

17. Bamberger E, Bamberger P. Unacceptable behaviours between healthcare workers: just the tip of the patient safety iceberg. BMJ Qual Saf. 2022;31:638–41.

18. Bambi S, Guazzini A, de Felippis C, Lucchini A, Rasero L. Preventing workplace incivility, lateral violence and bullying between nurses. A narrative literature review. Acta Biomedica. 2017;88:39–47.

19. Banerjee D, Nassikas NJ, Singh P, Andrea SB, Zhang AY, Aswad Y, et al. Feasibility of an Antiracism Curriculum in an Academic Pulmonary, Critical Care, and Sleep Medicine Division. Ats Scholar. 2022;3:433–48.

20. Barrett A, Piatek C, Korber S, Padula C. Lessons learned from a lateral violence and team-building intervention. Nursing Administration Quarterly. 2009;33:342–51.

21. Barzallo Salazar MJ, Minkoff H, Bayya J, Gillett B, Onoriode H, Weedon J, et al. Influence of surgeon behavior on trainee willingness to speak up: A randomized controlled trial. Journal of the American College of Surgeons. 2014;219:1001–7.

22. BBC News. NHS Highland pays out millions to bullied staff [Internet]. 2021. p. 1–6. Available from: <https://www.bbc.co.uk/news/uk-scotland-highlands-islands-58718290>

23. Beale D, Leather P. Working with care – improving working relationships in health and social care: self-assessment tools for health and social care teams [Internet]. Healthy workplace, healthy you. Dignity at work. Royal College of Nursing - RCN; 2005 p. 1–35. Available from: <https://www.rcn.org.uk/-/media/royal-college-of-nursing/documents/publications/2015/september/004972.pdf?la=en>

24. Benjamin A. Names, hair, identity and micro aggressions. British Medical Association. 2021;1–5.

25. Blackstock S, Cummings G, Glanfield F, Yonge O. A review: Developing an ecological model approach to co‐worker incivility experiences of new graduate nurses. Journal of Advanced Nursing. 2022;1–16.

26. Blackstock S, Salami B, Cummings GG. Organisational antecedents, policy and horizontal violence among nurses: An integrative review. Journal of Nursing Management. 2018;26:972–91.

27. Blakey AG, Anderson L, Smith-Han K, Wilkinson T, Collins E, Berryman E. Time to stop making things worse: An imperative focus for healthcare student bullying research. New Zealand Medical Journal. 2018;131:81–5.

28. British Medical Association. Workplace bullying and harassment of doctors A review of recent research [Internet]. British Medical Association. 2017. Available from: [file:///Users/VWC/Downloads/Bullying and harassment research review v7 WEB.pdf](https://doi.org/file:/Users/VWC/Downloads/Bullying%20and%20harassment%20research%20review%20v7%20WEB.pdf)

29. British Medical Association. Bullying and harassment : how to address it and create a supportive and inclusive culture. 2018.

30. British Medical Association. Promoting a positive working environment [Internet]. Advice and support. 2021 [cited 2021 Oct 20]. p. 1–8. Available from: <https://www.bma.org.uk/advice-and-support/discrimination-and-harassment/bullying-and-harassment/promoting-a-positive-working-environment>

31. Bry A, Wigert H. Organizational climate and interpersonal interactions among registered nurses in a neonatal intensive care unit: A qualitative study. Journal of Nursing Management (John Wiley & Sons, Inc). 2022;30:2031–8.

32. Carter M, Thompson N, Crampton P, Morrow G, Burford B, Gray C, et al. Workplace bullying in the UK NHS: A questionnaire and interview study on prevalence, impact and barriers to reporting. BMJ Open. 2013;3:1–12.

33. Ceravolo DJ, Schwartz DG, Foltz-Ramos KM, Castner J. Strengthening communication to overcome lateral violence. Journal of Nursing Management. 2012;20:599–606.

34. Chadwick S, Travaglia J. Workplace bullying in the Australian health context: a systematic review. Journal of Health, Organisation and Management. 2017;31:286–301.

35. Chipps EM, McRury M. The development of an educational intervention to address workplace bullying: A pilot study. Journal for Nurses in Staff Development. 2012;28:94–8.

36. Churchman JJ, Doherty C. Nurses’ views on challenging doctors’ practice in an acute hospital. Nursing standard (Royal College of Nursing (Great Britain) : 1987). 2010;24:42–7.

37. Churruca K, Pavithra A, McMullan R, Urwin R, Tippett S, Cunningham N, et al. Creating a culture of safety and respect through professional accountability: case study of the Ethos program across eight Australian hospitals. Australian Health Review. 2022;46:319–24.

38. Clark CM, Ahten SM, Macy R. Using Problem-Based Learning Scenarios to Prepare Nursing Students to Address Incivility. Clinical Simulation in Nursing. 2013;9:e75–83.

39. Colangelo A. St Vincent’s reviews anti-bullying program amid staff backlash. The Age. 2019;1–3.

40. Cooper K. Ending the silence [Internet]. BMA. 2018. Available from: <https://www.bma.org.uk/news-and-opinion/ending-the-silence>

41. Credland NJ, Whitfield C. Incidence and impact of incivility in paramedicine: A qualitative study. Emergency Medicine Journal. 2022;39:52–6.

42. Cruz D, Rodriguez Y, Mastropaolo C. Perceived microaggressions in health care: A measurement study. PLoS ONE. 2019;14:1–11.

43. Dahlby MA, Herrick LM. Evaluating an educational intervention on lateral violence. Journal of Continuing Education in Nursing. 2014;45:344–50.

44. Demarco RF, Roberts SJ, Chandler GE. The Use of a Writing Group to Enhance Voice and Connection Among Staff Nurses. Journal for Nurses in Professional Development. 2005;21:85–90.

45. Dimarino TJ. Eliminating Lateral Violence in the Ambulatory Setting: One Center’s Strategies. AORN Journal. 2011;93:583–8.

46. Dixon-Woods M, Campbell A, Martin G, Willars J, Tarrant C, Aveling EL, et al. Improving Employee Voice about Transgressive or Disruptive Behavior: A Case Study. Academic Medicine. 2019;94:579–85.

47. Edwards SL, O’Connell CF. Exploring bullying: Implications for nurse educators. Nurse Education in Practice. 2007;7:26–35.

48. Efe SY, Ayaz S. Mobbing against nurses in the workplace in Turkey. International Nursing Review. 2010;57:328–34.

49. Embree JL, Bruner DA, White A. Raising the Level of Awareness of Nurse-to-Nurse Lateral Violence in a Critical Access Hospital. Nursing Research and Practice. 2013;2013:1–7.

50. Felblinger DM. Bullying, incivility, and disruptive behaviors in the healthcare setting: identification, impact, and intervention. Frontiers of health services management. 2009;25:13–23.

51. Gamble Blakey A, Smith-Han K, Anderson L, Collins E, Berryman E, Wilkinson TJ. Interventions addressing student bullying in the clinical workplace: A narrative review. BMC Medical Education. 2019;19:1–13.

52. General Medical Council. Building a supportive environment: a review to tackle undermining and bullying in medical education and training. General Medical Council. 2015.

53. Gillespie GL, Grubb PL, Brown K, Boesch MC, Ulrich DL. “Nurses eat their young”: A novel bullying educational program for student nurses. Journal of Nursing Education and Practice. 2017;7:11.

54. Griffin M. Teaching cognitive rehearsal as a shield for lateral violence: an intervention for newly licensed nurses. Journal of continuing education in nursing. 2004;35:257–63.

55. Griffith M, Clery MJ, Humbert B, Joyce JM, Perry M, Hemphill RR, et al. Exploring Action Items to Address Resident Mistreatment through an Educational Workshop. The western journal of emergency medicine. 2019;21:42–6.

56. Hawkins N, Jeong S, Smith T. New graduate registered nurses’ exposure to negative workplace behaviour in the acute care setting: An integrative review. International Journal of Nursing Studies. 2019;93:41–54.

57. Hawkins N, Jeong SYS, Smith T, Sim J. A conflicted tribe under pressure: A qualitative study of negative workplace behaviour in nursing. Journal of Advanced Nursing. 2022;17:17–17.

58. Hawkins N, Jeong SYS, Smith T, Sim J. Creating respectful workplaces for nurses in regional acute care settings: A quasi-experimental design. Nursing Open. 2022;78–89.

59. Hemmings N, Buckingham H, Oung C, Palmer W. Attracting, supporting and retaining a diverse NHS workforce [Internet]. 2021. Available from: [www.nuffieldtrust.org.uk/research](https://doi.org/www.nuffieldtrust.org.uk/research)

60. Hickson GB, Pichert JW, Webb LE, Gabbe SG. A complementary approach to promoting professionalism: Identifying, measuring, and addressing unprofessional behaviors. Academic Medicine. 2007;82:1040–8.

61. Hughes A. Being bullied what an insight. British journal of perioperative nursing : the journal of the National Association of Theatre Nurses. 2003;13:166–72.

62. Hutchinson M, Jackson D, Wilkes L, Vickers MH. A new model of bullying in the nursing workplace organizational characteristics as critical antecedents. Advances in Nursing Science. 2008;31:60–71.

63. Hutchinson M, Wilkes L, Jackson D, Vickers MH. Integrating individual, work group and organizational factors: Testing a multidimensional model of bullying in the nursing workplace. Journal of Nursing Management. 2010;18:173–81.

64. Illing J, Carter M, Thompson NJ, Crampton PES, Morrow GM, Howse JH, et al. Evidence synthesis on the occurrence, causes, management of bullying and harassing behaviours to inform decision making in the NHS. Final report. NIHR Service Delivery and Organisation Programme. 2013 p. 1–265. Report No.: February.

65. Illing J, Thompson N, Crampton P, Charlotte M, Ms R, Kehoe A, et al. Workplace bullying: measurements and metrics to use in the NHS Final Report for NHS Employers [Internet]. Newcastle University; 2016 p. 1–58. Report No.: March. Available from: [http://www.nhsemployers.org/$\sim$/media/Employers/Documents/Campaigns/NHS_Employers_Bullying_Measures_Final_Report.pdf](http://www.nhsemployers.org/$/sim$/media/Employers/Documents/Campaigns/NHS_Employers_Bullying_Measures_Final_Report.pdf)

66. Işık I, Gümüşkaya O, Şen S, Arslan Özkan H. The Elephant in the Room: Nurses’ Views of Communication Failure and Recommendations for Improvement in Perioperative Care. AORN Journal. 2020;111:e1–15.

67. Jenkins S, Woith W, Kerber C, Stenger D. Why can’t we all just get along? A civility journal club intervention. Nurse Educator. 2011;36:140–1.

68. Johnson MJ, May CR. Promoting professional behaviour change in healthcare: What interventions work, and why? A theory-led overview of systematic reviews. BMJ Open. 2015;5.

69. Johnson SL, Haerling KA, Yuwen W, Huynh V, Le C. Incivility and Clinical Performance, Teamwork, and Emotions: A Randomized Controlled Trial. Journal of Nursing Care Quality. 2020;35:70–6.

70. Jones A, Kelly D. Deafening silence? Time to reconsider whether organisations are silent or deaf when things go wrong. BMJ Quality and Safety. 2014;23:709–13.

71. Kaiser JA. The relationship between leadership style and nurse-to-nurse incivility: turning the lens inward. Journal of Nursing Management. 2017;25:110–8.

72. Kang J, Jeong YJ. Effects of a smartphone application for cognitive rehearsal intervention on workplace bullying and turnover intention among nurses. International Journal of Nursing Practice. 2019;25:1–10.

73. Kang J, Kim JI, Yun S. Effects of a cognitive rehearsal program on interpersonal relationships, workplace bullying, symptom experience, and turnover intention among nurses: A randomized controlled trial. Journal of Korean Academy of Nursing. 2017;47:689–99.

74. Keller S, Yule S, Zagarese V, Parker SH. Predictors and triggers of incivility within healthcare teams: A systematic review of the literature. BMJ Open. 2020;10:1–15.

75. Kile D, Eaton M, DeValpine M, Gilbert R. The effectiveness of education and cognitive rehearsal in managing nurse-to-nurse incivility: A pilot study. Journal of Nursing Management. 2019;27:543–52.

76. Kline R. Racism which impacts healthcare staff endangers patient care [Internet]. Middlesex University London. 2021. Available from: <https://mdxminds.com/2021/11/22/racism-which-impacts-healthcare-staff-endangers-patient-care/>

77. Kline R. A review into culture and bullying at University Hospitals of North Midlands NHS Trust. 2022;

78. Kousha S, Shahrami A, Forouzanfar MM, Sanaie N, Atashzadeh-Shoorideh F, Skerrett V. Effectiveness of educational intervention and cognitive rehearsal on perceived incivility among emergency nurses: a randomized controlled trial. BMC Nursing. 2022;21:153–153.

79. Lasater K, Mood L, Buchwach D, Dieckmann NF. Reducing incivility in the workplace: Results of a three-part educational intervention. Journal of Continuing Education in Nursing. 2015;46:15–24.

80. Laschinger HKS, Leiter MP, Day A, Gilin-Oore D, MacKinnon SP. Building empowering work environments that foster civility and organizational trust: Testing an intervention. Nursing Research. 2012;61:316–25.

81. Leiter MP, Laschinger HKS, Day A, Oore DG. The impact of civility interventions on employee social behavior, distress, and attitudes. Journal of Applied Psychology. 2011;96:1258–74.

82. Lewis D. Workplace Culture at Southwestern Ambulance NHS Foundation Trust. 2018.

83. Longo J, Hain D. Bullying: a hidden threat to patient safety. Nephrology nursing journal : journal of the American Nephrology Nurses’ Association. 2014;41:193–9; quiz 200.

84. Lovejoy-Bluem A. Incivility and/or Human Kind(ness) in the NICU. Academy of Neonatal Nursing. 2016;35:173–4.

85. Maben J, Adams M, Peccei R, Murrells T, Robert G. “Poppets and parcels”: The links between staff experience of work and acutely ill older peoples’ experience of hospital care. International Journal of Older People Nursing. 2012;7:83–94.

86. Mannion R, Davies H, Powell M, Blenkinsopp J, Millar R, McHale J, et al. Healthcare scandals and the failings of doctors: Do official inquiries hold the profession to account? Journal of Health Organization and Management. 2019;33:221–40.

87. Manton AP. Bullying: A Pebble in the Pond. Journal of Emergency Nursing. 2017;43:389–90.

88. Markwell A, Smith S, Michalski M, Conroy S, Bell A. Performance management versus bullying and harassment: An educator perspective. EMA - Emergency Medicine Australasia. 2015;27:468–72.

89. McKenzie LN, Shaw L, Jordan JE, Alexander M, O’Brien M, Singer SJ, et al. Factors Influencing the Implementation of a Hospitalwide Intervention to Promote Professionalism and Build a Safety Culture: A Qualitative Study. Joint Commission Journal on Quality and Patient Safety. 2019;45:694–705.

90. Mello MM, Jagsi R. Standing Up against Gender Bias and Harassment — A Matter of Professional Ethics. New England Journal of Medicine. 2020;6:510–2.

91. Miller DT, Chen EH. Helping the learner to deal with microaggressions in the workplace: Individual, programmatic, and institutional-level responses. AEM Education and Training. 2021;5:S140–3.

92. Mitchell G. Bullying and inadequate leadership found at flagship nursing trust. Nursing Times. 2021;1–6.

93. National Freedom to Speak Up Guardian. Bullying behaviour is unacceptable. It is unprofessional and unneccessary. It affects the wellbeing of individuals and the teams within which they work. 2018.

94. National Guardian’s Office. Speaking up in the NHS in England: A summary of speaking up to Freedom to Speak Up Guardians in NHS trusts and foundation trusts. 2018. Report No.: September 2018.

95. Naylor MJ, Boyes C, Killingback C. “You’ve broken the patient”: Physiotherapists’ lived experience of incivility within the healthcare team - An Interpretative Phenomenological Analysis. Physiotherapy. 2022;117:89–96.

96. NHS Employers. Tackling bullying in ambulance trusts: a guide for action. 2016;

97. Nicotera AM, Mahon MM, Wright KB. Communication that builds teams: Assessing a nursing conflict intervention. Nursing Administration Quarterly. 2014;38:248–60.

98. Nikstaitis T, Simko LC. Incivility among intensive care nurses: The effects of an educational intervention. Dimensions of Critical Care Nursing. 2014;33:293–301.

99. Nursing & Midwifery Council. The NMC register. 2021. Report No.: April 2020.

100. O’Connell KM, Garbark RL, Nader KC. Cognitive Rehearsal Training to Prevent Lateral Violence in a Military Medical Facility. Journal of Perianesthesia Nursing. 2019;34:645-653.e1.

101. O’Keeffe DA, Brennan SR, Doherty EM. Resident Training for Successful Professional Interactions. Journal of Surgical Education. 2022;79:107–11.

102. Osatuke K, Moore SC, Ward C, Dyrenforth SR, Belton L. Civility, Respect, Engagement in the Workforce (CREW). The Journal of Applied Behavioral Science. 2009;45:384–410.

103. Owens J, Singh G, Cribb A. Austerity and Professionalism: Being a Good Healthcare Professional in Bad Conditions. Health Care Analysis. 2019;27:157–70.

104. Parizad N, Hassankhani H, Rahmani A, Mohammadi E, Lopez V, Cleary M. Nurses’ experiences of unprofessional behaviors in the emergency department: A qualitative study. Nursing and Health Sciences. 2018;20:54–9.

105. Parker KM, Harrington A, Smith CM, Sellers KF, Millenbach L. Creating a Nurse-Led Culture to Minimize Horizontal Violence in the Acute Care Setting: A Multi-Interventional Approach. Journal for Nurses in Professional Development. 2016;32:56–63.

106. Pavithra A, Sunderland N, Callen J, Westbrook J. Unprofessional behaviours experienced by hospital staff: qualitative analysis of narrative comments in a longitudinal survey across seven hospitals in Australia. BMC Health Services Research. 2022;1–15.

107. Phillips JM, Stalter AM, Winegardner S, Wiggs C, Jauch A. Systems thinking and incivility in nursing practice: An integrative review. Nursing Forum. 2018;53:286–98.

108. Pisklakov S, Tilak V, Patel A, Xiong M. Bullying and Aggressive Behavior among Health Care Providers: Literature Review. Advances in Anthropology. 2013;03:179–82.

109. Purpora C, Blegen MA. Horizontal Violence and the Quality and Safety of Patient Care: A Conceptual Model. Nursing Research and Practice. 2012;2012:1–5.

110. Quinlan E, Robertson S, Miller N, Robertson-Boersma D. Interventions to reduce bullying in health care organizations: A scoping review. Health Services Management Research. 2014;27:33–44.

111. Riskin A, Erez A, Foulk TA, Kugelman A, Gover A, Shoris I, et al. The impact of rudeness on medical team performance: A randomized trial. Pediatrics. 2015;136:487–95.

112. Riskin A, Erez A, Foulk TA, Riskin-Geuz KS, Ziv A, Sela R, et al. Rudeness and medical team performance. Pediatrics. 2017;139:1–11.

113. Rocker CF. Addressing nurse-to-nurse bullying to promote nurse retention. Online Journal of Issues in Nursing. 2008;13:1–10.

114. Rogers-Clark C, Pearce S, Cameron M. Management of disruptive behaviour within nursing work environments: a comprehensive systematic review of the evidence. JBI Library of Systematic Reviews. 2009;7:615–78.

115. Ross S, Jabbal J, Chauhan K, Maguire D, Randhawa M, Dahir S. Workforce race inequalities and inclusion in NHS providers. 2020;

116. Royal College of Nursing. Bullying and harassment: good practice guidance for preventing and addressing bullying and harassment in health and social care organisations. 2014 p. 22–4.

117. Royal College of Surgeons of England. MANAGING DISRUPTIVE BEHAVIOURS IN SURGERY: A Guide to Good Practice [Internet]. 2021. Available from: [www.rcseng.ac.uk/standardsandguidance](https://doi.org/www.rcseng.ac.uk/standardsandguidance)

118. Rutherford DE, Gillespie GL, Smith CR. Interventions against bullying of prelicensure students and nursing professionals: An integrative review. Nursing Forum. 2019;54:84–90.

119. Salin D. Ways of explaining workplace bullying: A review of enabling, motivating and precipitating structures and processes in the work environment. Human Relations. 2003;56:1213–32.

120. Saxton R. Communication Skills Training to Address Disruptive Physician Behavior. AORN Journal. 2012;95:602–11.

121. Sheehan M, McCabe TJ, Garavan TN. Workplace bullying and employee outcomes: a moderated mediated model. International Journal of Human Resource Management. 2020;31:1379–416.

122. Shuttleworth A. Can Arrowe Park show other trusts how to beat the bullies? Nursing Times. 2018;114:12–12.

123. Sillero AS, Buil N. Enhancing interprofessional collaboration in perioperative setting from the qualitative perspectives of physicians and nurses. International Journal of Environmental Research and Public Health. 2021;18:1–11.

124. Solheim J. Caring for Each Other While We Care for Others. Journal of Emergency Nursing. 2018;44:319–20.

125. Speck RM, Foster JJ, Mulhern VA, Burke SV, Sullivan PG, Fleisher LA. Development of a professionalism committee approach to address unprofessional medical staff behavior at an academic medical center. Joint Commission Journal on Quality and Patient Safety. 2014;40:161–7.

126. Stagg SJ, Sheridan DJ, Jones RA, Speroni KG. Workplace Bullying: The Effectiveness of a Workplace Program. Australian nursing & midwifery journal. 2017;24:34–6.

127. Stagg SJ, Sheridan D, Jones RA, Speroni KG. Evaluation of a Workplace Bullying Cognitive Rehearsal Program in a Hospital Setting. The Journal of Continuing Education in Nursing. 2011;42:395–403.

128. Stevens S. Nursing workforce retention: Challenging a bullying culture. Health Affairs. 2002;21:189–93.

129. Stone L, Phillips C, Douglas KA. Sexual assault and harassment of doctors, by doctors: a qualitative study. Medical Education. 2019;53:833–43.

130. Tame S. The relationship between continuing professional education and horizontal violence in perioperative practice. Journal of perioperative practice. 2012;22:220–5.

131. Taylor RA, Taylor SS. Reframing and addressing horizontal violence as a workplace quality improvement concern. Nursing Forum. 2018;53:459–65.

132. Thorsness R, Sayers B. Systems Approach to Resolving Conduct Issues Among Staff Members. AORN Journal. 1995;61:197–202.

133. Tran V. Dealing with bullying and harassment: A practical guide for Australasian emergency medicine trainees. EMA - Emergency Medicine Australasia. 2015;27:473–7.

134. Tuffour I. It is like ‘judging a book by its cover’: An exploration of the lived experiences of Black African mental health nurses in England. Nursing Inquiry. 2022;29:1–12.

135. Venkatesh B, Corke C, Raper R, Pinder M, Stephens D, Joynt G, et al. Findings of the bullying, discrimination and sexual harassment survey: Response of the college of intensive care medicine. Critical Care and Resuscitation. 2016;18:228–9.

136. Villafranca A, Hamlin C, Enns S, Jacobsohn E. Disruptive behaviour in the perioperative setting: a contemporary review. Canadian Journal of Anesthesia. 2017;64:128–40.

137. Walton MM. Hierarchies: The Berlin wall of patient safety. Quality and Safety in Health Care. 2006;15:229–30.

138. Warrner J, Sommers K, Zappa M, Thornlow DK. Decreasing work place incivility. Nursing Management. 2016;47:22–30.

139. Weaver KB. The effects of horizontal violence and bullying on new nurse retention. Journal for Nurses in Professional Development. 2013;29:138–42.

140. Webb LE, Dmochowski RR, Moore IN, Pichert JW, Catron TF, Troyer M, et al. Using coworker observations to promote accountability for disrespectful and unsafe behaviors by physicians and advanced practice professionals. Joint Commission Journal on Quality and Patient Safety. 2016;42:149–61.

141. Westbrook JI, Urwin R, McMullan R, Badgery-Parker T, Pavithra A, Churruca K, et al. Changes in the prevalence of unprofessional behaviours by co-workers following a professional accountability culture change program across five Australian hospitals. 2023;

142. Westbrook J, Sunderland N, Atkinson V, Jones C, Braithwaite J. Endemic unprofessional behaviour in health care: the mandate for a change in approach. The Medical journal of Australia. 2018;209:380–1.

143. Wild JRL, Ferguson HJM, McDermott FD, Hornby ST, Gokani VJ. Undermining and bullying in surgical training: A review and recommendations by the Association of Surgeons in Training. International Journal of Surgery. 2015;23:S5–9.

144. Wilson JL. An exploration of bullying behaviours in nursing: a review of the literature. British Journal of Nursing. 2016;25:303–6.

145. Yu F, Raphael D, Mackay L, Smith M, King A. Personal and work-related factors associated with nurse resilience: A systematic review. International Journal of Nursing Studies. 2019;93:129–40.

146. Zhang X, Xiong L. Impact of Nurse Horizontal Violence and Coping Strategies: A Review. Yangtze Medicine. 2019;03:289–300.
